# Supplementary material for: DDB1 engagement defines the selectivity of S656 analogs for cyclin K degradation over CDK inhibition
Source: EMBO Rep. 2025 Apr 28;26(11):2836–54. doi: 10.1038/s44319-025-00448-y (PMC12152147; doi:10.1038/s44319-025-00448-y)
Supplement: Supplementary file 1 — Appendix [file 44319_2025_448_MOESM1_ESM.pdf]

Appendix

DDB1 engagement defines the selectivity of S656 analogs for cyclin K degradation over CDK inhibition

Moison *et al.*

Table of content

Appendix Figures S1 ………………………………………………………. Page 2
Appendix Figures S2 ………………………………………………………. Page 3
Appendix Figures S3 ………………………………………………………. Page 4
Appendix Figures S4 ………………………………………………………. Page 5
Appendix Figures S5 ………………………………………………………. Page 6
Appendix Figures S6 ………………………………………………………. Page 7
Appendix Figures S7 ………………………………………………………. Page 9
Synthetic chemistry supporting information ……………………………..Page 11

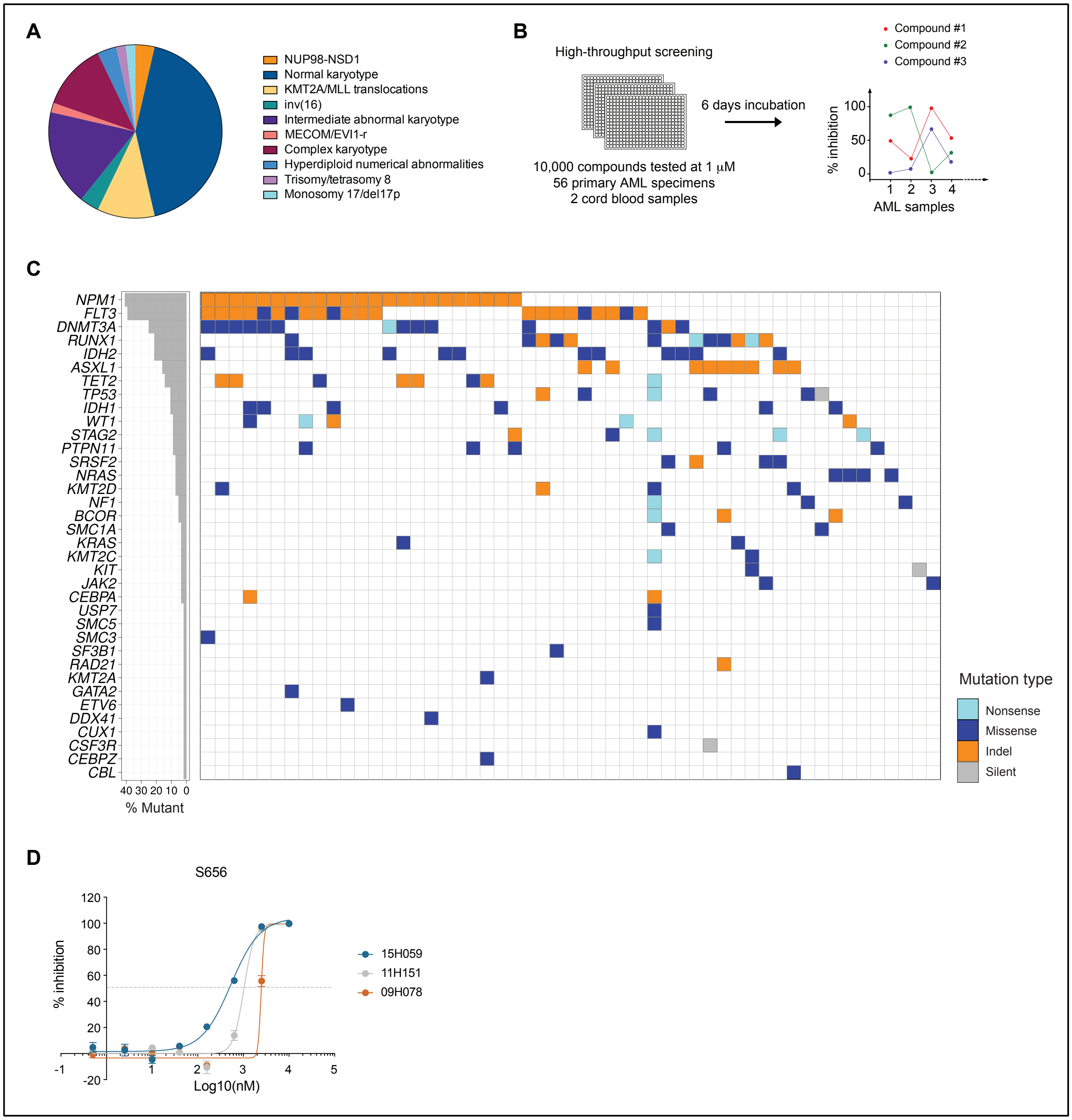

Appendix Figure S1: (A) AML subtype classification of the 56 primary specimens used in discovery screen. (B) High-throughput screening strategy. (C) Mutation heatmap of the 56 primary AML used in discovery screen (3 specimens without mutations are not represented). Genes (y-axis) are ordered based on their mutation frequencies while AML specimens are represented in the x-axis. (D) Representative dose-response curves for S656 obtained in 3 primary AML samples from the validation screen (error bars indicate SD of technical duplicates). Discovery screen (panels A to C) has been performed and described previously (Moison *et al*, 2024).

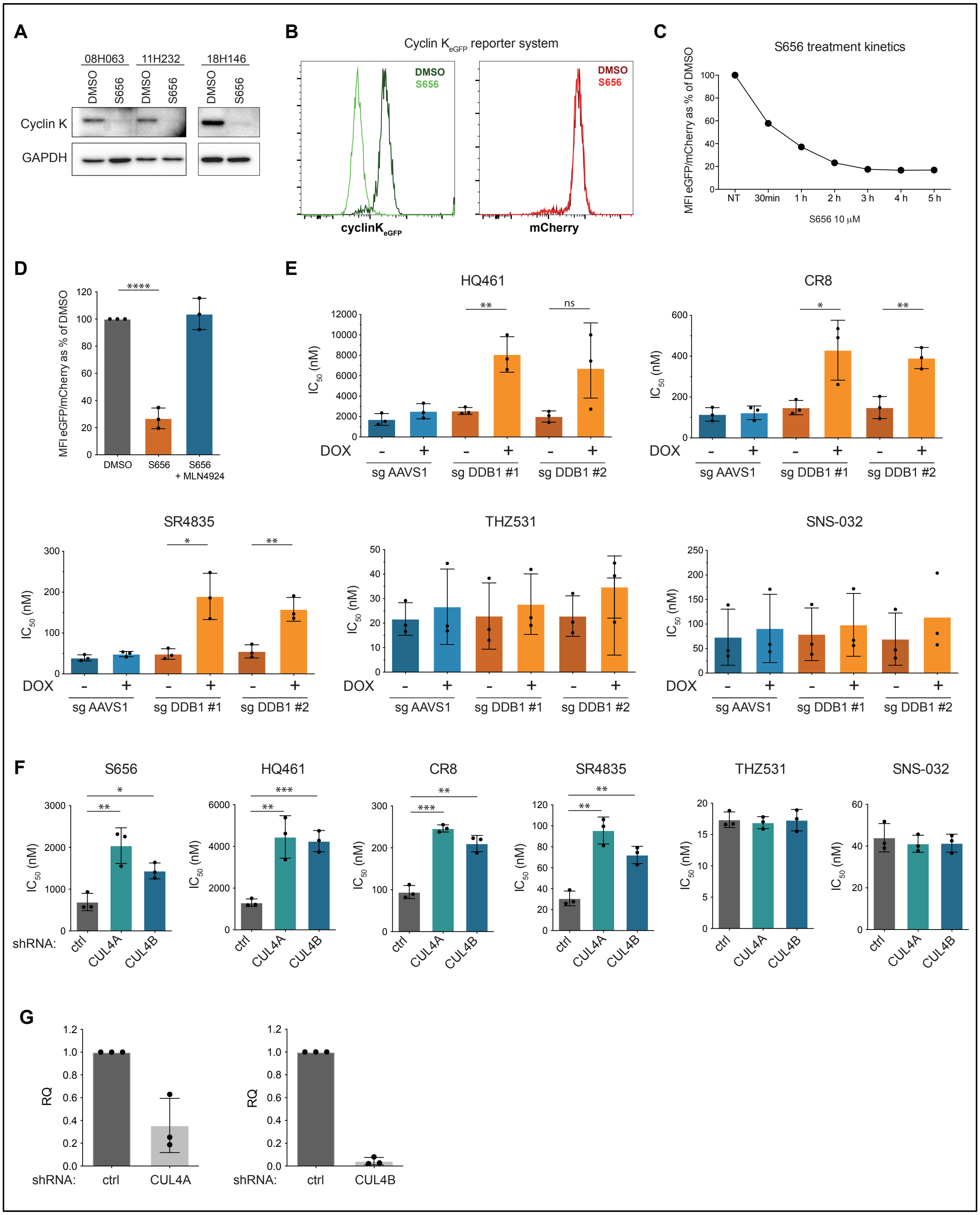

Appendix Figure S2: (A) Immunoblot analysis of cyclin K in 3 primary AML specimens, after exposure to DMSO or S656 (5 hours at 5 μM). GAPDH is used as a loading control. (B) Flow cytometry profile of eGFP and mcherry fluorescence intensity in OCI-AML5 G7 clone stably

expressing the cyclin K reporter system. The decrease of eGFP fluorescence in cells treated for 3 hours with 10 µM S656 reflects cyclin K_{eGFP} degradation. mcherry fluorescence is used as an internal control. (C) Kinetics of cyclin K_{eGFP} fluorescence upon exposure to 10 µM of S656 in OCI-AML5 G7 clone. Results are normalized to fluorescence in DMSO-treated cells. MFI = Mean Fluorescence Intensity. (D) Cyclin K degradation assessment by measuring the MFI of cyclin K_{eGFP} over mCherry by flow cytometry in OCI-AML5 G7 clone exposed 3 hours to 10 µM of S656 with or without 1 hr pre-incubation with 2.5 µM of MLN4924 (mean +/- SD, n=3, biological replicates, t-test). (E) Dose response experiment to determine IC_{50} values of known cyclin K degraders HQ461, CR8 and SR4835, as well as the CDK12/13 inhibitor THZ531 and the CDK2/7/9 inhibitor SNS-032, in inducible (+DOX) AML5 Cas9 cells expressing two different sgRNAs targeting *DDB1* gene or control region *AAVS1* (4 days incubation, mean +/- SD, n=3, biological replicates, t-test). (F) Dose response experiment to determine IC_{50} values of S656, known cyclin K degraders (HQ461, CR8 and SR4835) and CDK inhibitors (THZ531 and SNS-032) in OCI-AML5 cells expressing shRNAs targeting *CUL4A*, *CUL4B* or control sequence (4 days incubation, mean +/- SD, n=3, biological replicates, t-test). (G) Assessment of *CUL4A* and *CUL4B* mRNA downregulation by qPCR in OCI-AML5 cells. RNA was collected 72 hours after infection with respective shRNAs (mean +/- SD, n=3, biological replicates). Normalized to *HPRT*.

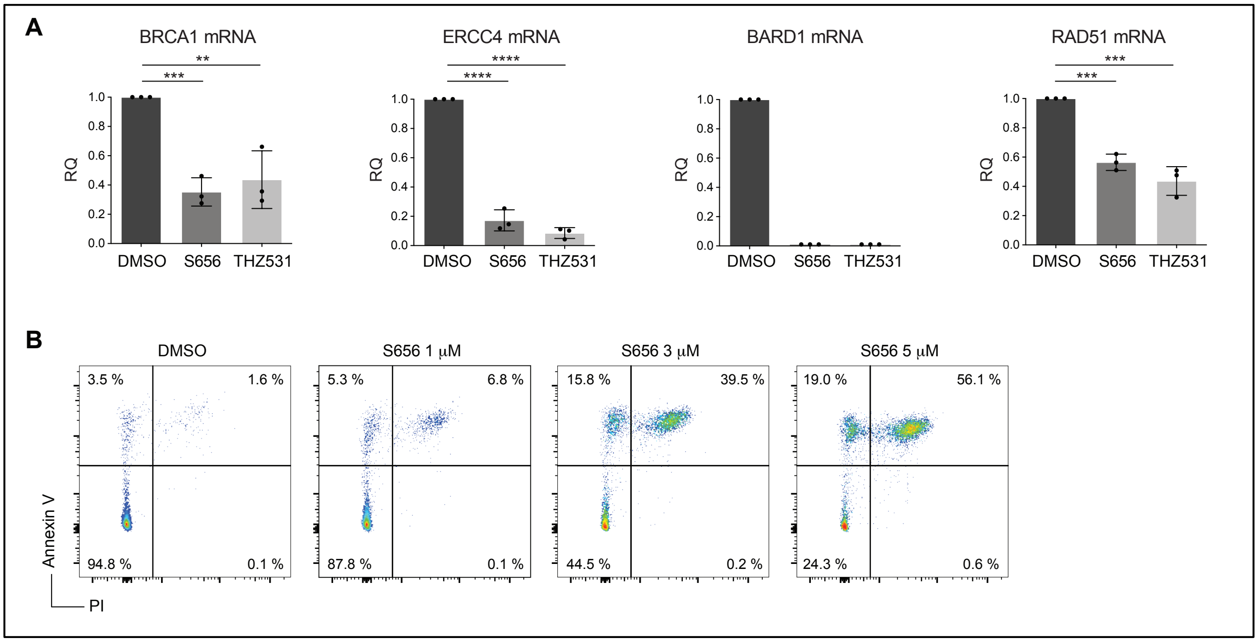

<u>Appendix Figure S3:</u> (A) Monitoring of *ERCC4, BARD1*, *BRCA1* and *RAD51* mRNA expression by qPCR on OCI-AML5 cells treated 4 hours with 5 µM of S656 or 200 nM of the CDK12/13 inhibitor THZ531. Normalized to *HPRT* (mean +/- SD, n=3, biological replicates, t-test). (B) Representative cytometry profiles of Annexin V / PI staining of OCI-AML5 cells treated for 24 hours with increasing concentrations of S656.

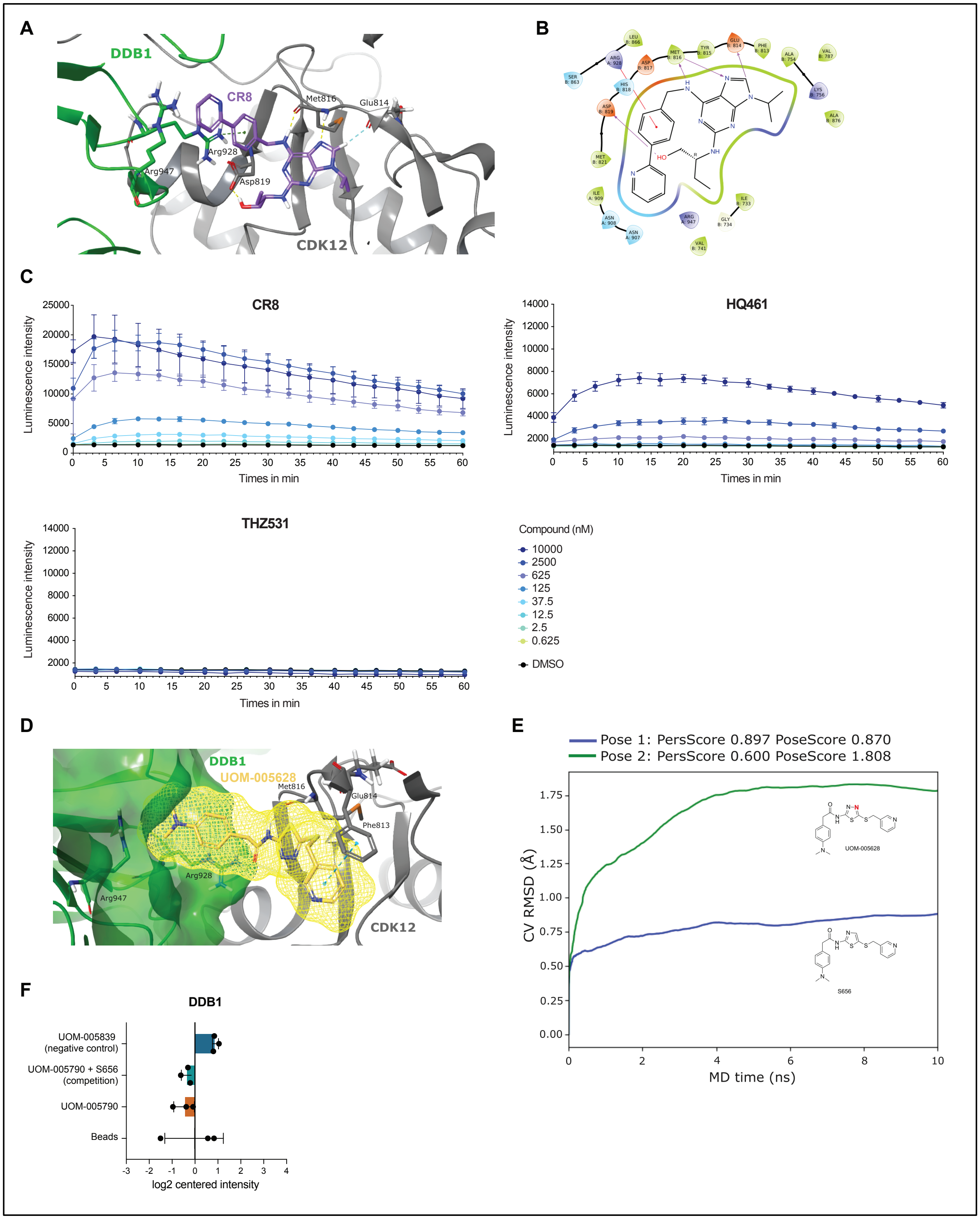

Appendix Figure S4: (A) 3D representation of CR8 (in purple, stick representation) bound to CDK12 (grey ribbons) and DDB1 (green ribbons). Interactions with the hinge region of CDK12 are shown: Met816 provides two backbone interactions, a hydrogen bond donor, and a hydrogen

bond acceptor, respectively while the CH on the imidazole of CR8 participates in an aromatic H-bond with Glu814 backbone carbonyl. CR8 also shows interaction with Asp819 sidechain on CDK12 (hydrogen bonds: yellow dashed line, aromatic H-bonds: turquoise dashed line). The phenyl ring of CR8 establishes a cation-π interaction with Arg928 on DDB1, shown in green dashed line. (B) 2D interaction diagram of CR8 bound to CDK12 and DDB1, in which purple arrows represent H-bonds and the red line stands for a cation-π interaction. (C) Luminescence intensity generated by the reconstitution of the NanoLuc was monitored over 1 hour right after the addition of CR8, HQ461 and THZ531 compounds at increasing concentrations in HEK293 cells (mean +/- SD, n=4, biological replicates). (D) 3D representation of UOM-005628 (in yellow, stick representation) bound to CDK12 (grey ribbons) and DDB1 (green surface representation). The van der Waals surface of UOM-005628 is shown in yellow Mesh to showcase the volume of the molecule. Interactions with the hinge region of CDK12 are established: Met816 provides one backbone interaction, a hydrogen bond acceptor, interacting with the NH on compound UOM-005628. UOM-005628's pyridine ring engages in π-π stacking with Phe813. The phenyl ring of UOM-005628 establishes a cation-π interaction with Arg928 on DDB1, shown in green dashed lines (hydrogen bonds: yellow dashed line, aromatic H-bonds: turquoise dashed line). The docked pose suggests a rotation of the thiadiazole when compared to the thiazole present in S656, as the hinge binder motif is not present in this compound. (E) Binding pose Metadynamics (BPMD) comparing the stability of S656 and UOM-005628 in the binding site. The key concept in BPMD is that under the same biasing force, ligands that are not stably bound to the receptor will experience a higher fluctuation in their RMSD (Root Mean Square Deviation) as compared to those stably bound. Therefore, ligand poses which are unstable under the bias of those metadynamics simulation are not or rarely expected to be occupied in the energy landscape. S656 exhibits very stable binding (blue line), with an average RMSD of around 0.6-0.7 Angstrom while UOM-005628 (green line) exhibits fluctuation of its RMSD around 1,75 Angstrom. Binding pose Meta Dynamics were calculated 10 x 10ns using Desmond (see Methods), an average of all runs is shown. (F) Graphical representation of the pull-down enrichments obtained for DDB1 using the specific UOM-005790 probe (+/- competition with S656) and the negative control probe UOM-005839 (mean +/- SD, n=3, biological replicates).

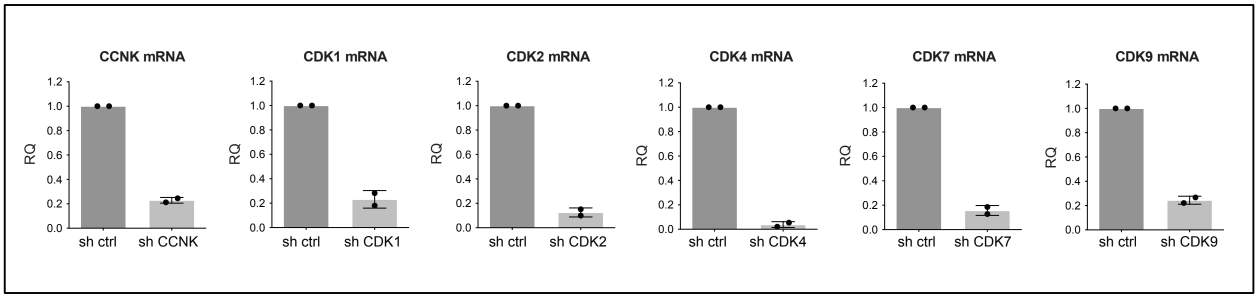

<u>Appendix Figure S5:</u> Assessment of *CCNK*, *CDK1, 2, 4, 7* and *9* mRNA downregulation by qPCR in OCI-AML5 cells expressing the corresponding shRNAs. Normalized to *HPRT* (n=2, biological replicates).

Class I - Cyclin K degraders

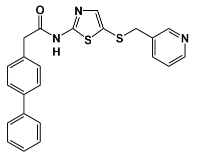

UOM-005429

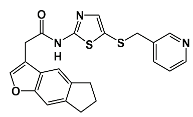

UOM-005479

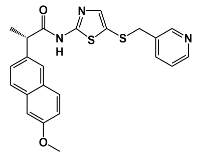

UOM-005431

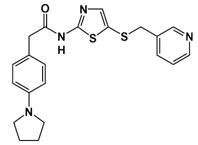

UOM-005608

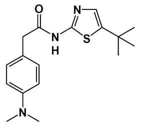

UOM-005605

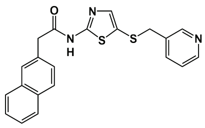

UOM-005428

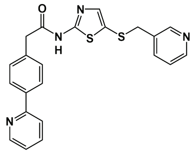

UOM-005636

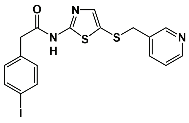

UOM-005200

Mixed Class I-II

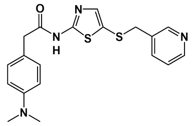

S656

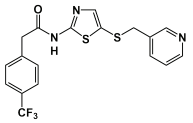

UOM-005203

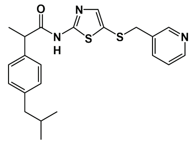

UOM-005430

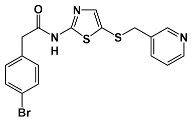

UOM-005199

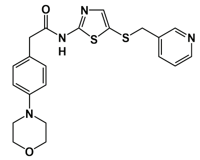

UOM-005606

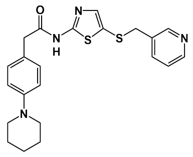

UOM-005607

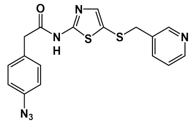

UOM-005062

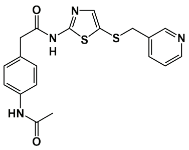

UOM-005473

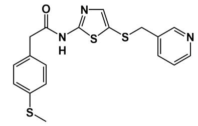

UOM-005206

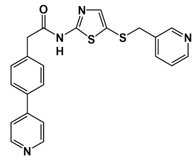

UOM-005550

Inactive

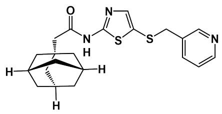

UOM-005433

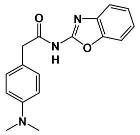

UOM-005604

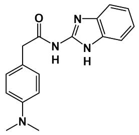

UOM-005603

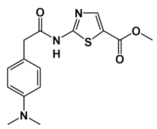

UOM-005602

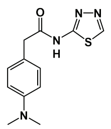

UOM-005601

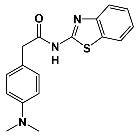

UOM-005600

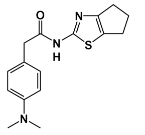

UOM-005599

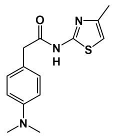

UOM-005598

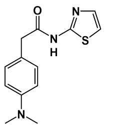

UOM-005597

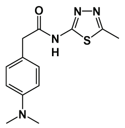

UOM-005596

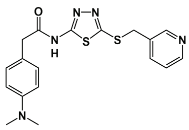

UOM-005628

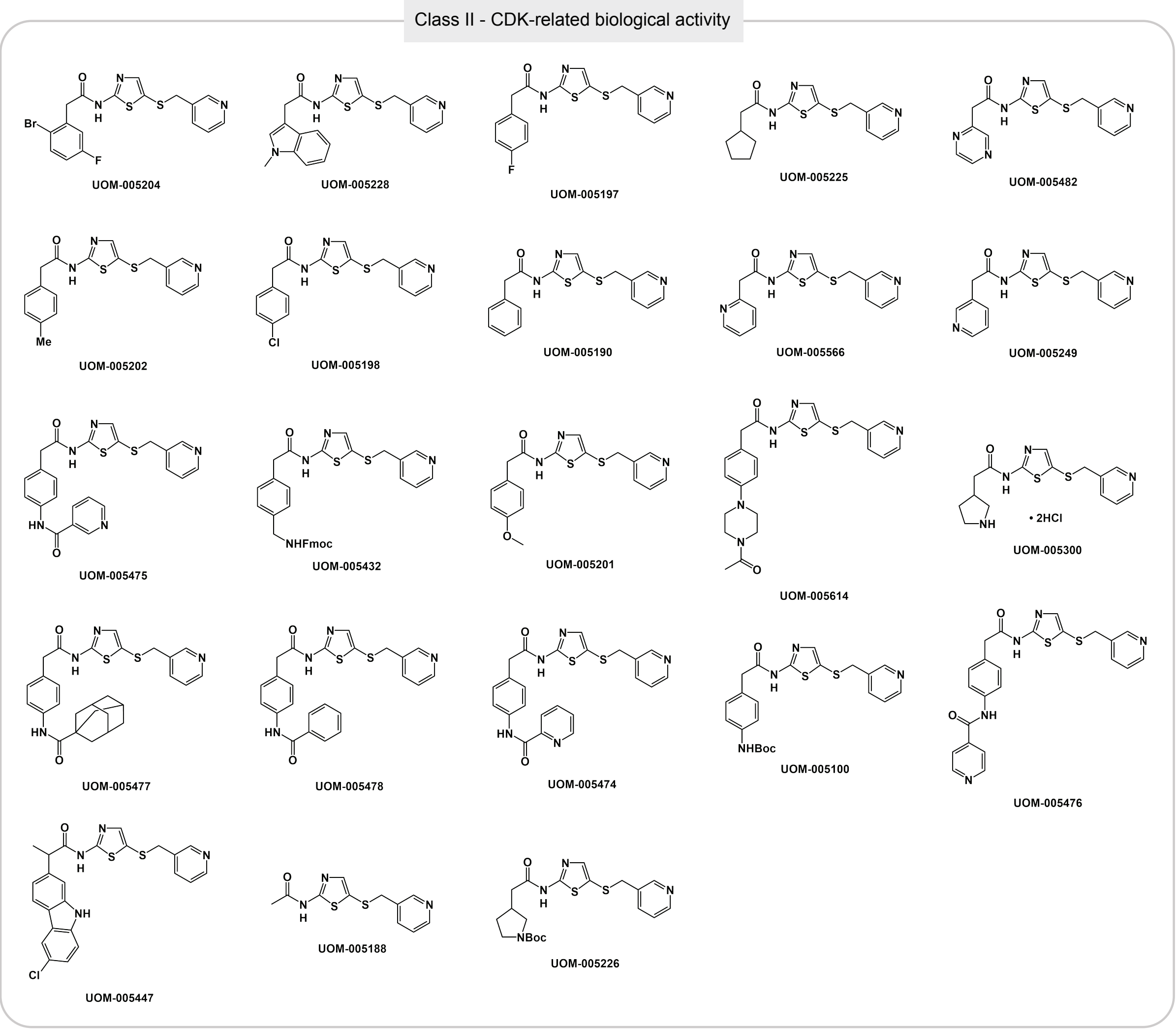

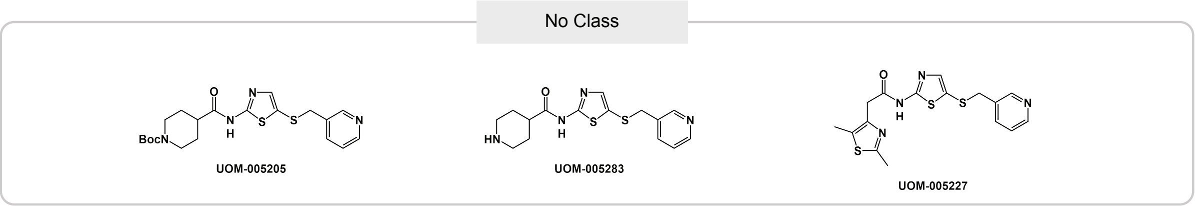

<u>Appendix Figure S6:</u> Structures and classification of S656 analogs.

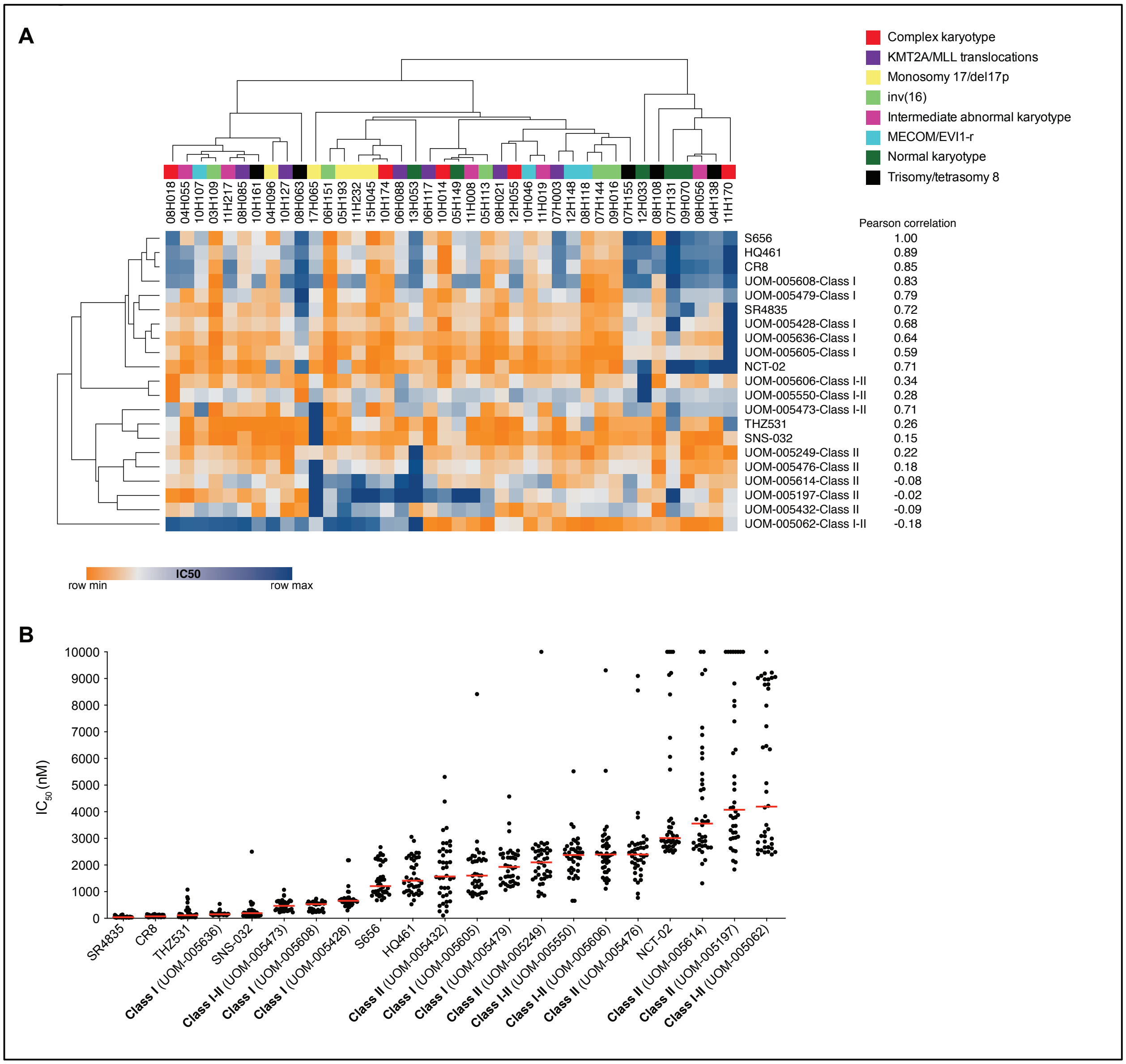

Appendix Figure S7: (A) Heat map representation and hierarchical clustering of IC_{50} values obtained from selected Class I, I-II and II compounds along with controls across 40 primary AML specimens. Correlation between S656 and the other molecules is indicated. Relative color scheme uses the minimum and maximum IC_{50} values in each row. (B) IC_{50} values and median (red) of all compounds tested in a panel of 40 primary AMLs as shown in panel A and Figure 4. Compounds were ordered by median potency.

REFERENCES

Moison C, Gracias D, Schmitt J, Girard S, Spinella J-F, Fortier S, Boivin I, Mendoza-Sanchez R, Thavonekham B, MacRae T, *et al* (2024) SF3B1 mutations provide genetic vulnerability to copper ionophores in human acute myeloid leukemia. *Sci Adv* 10: eadl4018

Synthetic chemistry supporting information

Table of contents

General experimental information 11
Synthesis of synthetic intermediates II, V, IX, XII, and XV 12
Synthesis aminothiazole II 12
Synthesis of amino acid V 13
Synthesis of carboxylic acid IX 14
Synthesis of carboxylic acid XII 15
Synthesis of aniline XV 16
Synthesis of carboxylic acid XVII 17
Synthesis of S656 derivatives 18
References 38

General experimental information

All commercially available chemicals and solvents were used, unless specified, without further purification. Reactions were performed under an atmosphere of nitrogen. Organic solutions were concentrated under reduced pressure on a rotary evaporator using a water bath. Reactions were monitored *via* LC-MS on an Agilent 1260 Infinity system paired with a single-quad mass spectrometer that gave all the low-resolution mass spectra (ESI) collected for this document. Normal phase chromatography was performed on Santai Tech SepaFlash™ prepacked cartridges (particle size of 40-63 μm) and reversed-phase chromatography was performed on Biotage® Sfär C18 Duo columns (particle size of 30 μm). Both normal and reversed phase chromatographic purifications were carried out on Teledyne Isco CombiFlash systems with gradients of the specified solvents. Preparative reverse-phase high-pressure liquid chromatography was carried out on an Agilent 1200 series HPLC using or on a Teledyne ISCO Combliflash EZ Prep on Kinetex® C18 columns (100 x 30 mm) with a 5 μm particle size and using methanol/water gradients containing 0.1% formic acid unless otherwise stated. Analytical purity of compounds was obtained on an Agilent 1200 series HPLC on Poroshell 120 C18, 2.7 μm, 50 x 4.6 mm column at room temperature with a flow of 1.25 mL/min. The gradient consisted of elutents A (0.1% formic acid in double distilled water) and B (0.1% formic acid in HPLC-grade methanol). Absorbance was monitored at λ=214 nm and λ=254 nm. The gradient method started at 5% of B and increased from 5% to 95% B in 30 sec followed by 2 min at 95% of B. ^{1}H NMR was obtained on a Bruker Ascend 400 MHz and the recorded shifts for protons are reported in parts per million (δ scale) downfield from tetramethylsilane and are referenced to residual protium in the NMR solvents ($CDCl_3$: δ 7.26, DMSO: δ 2.50, centre line). Data are represented as follows: chemical shift δ in

ppm, multiplicity (s singlet, d doublet, t triplet, q quartet, m multiplet, br broad, etc.), coupling constant J in Hz and integration.

Synthesis of synthetic intermediates II, V, IX, XII, and XV

Synthesis aminothiazole II

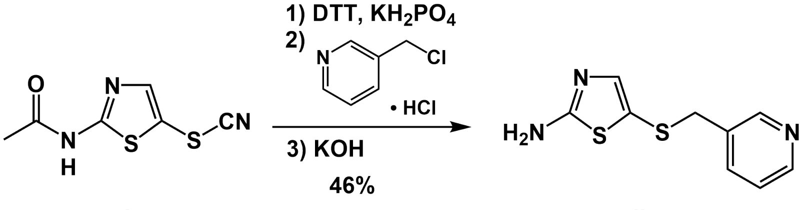

5-((pyridin-3-ylmethyl)thio)thiazol-2-amine (**I**). In a 1 L RBF, N-(5-thiocyanatothiazol-2-yl)acetamide (I)(Kim *et al*, 2002) (1 eq, 6.9 g, 34.63 mmol) was dissolved in deoxygenated methanol (280 mL) and a 2.0M solution of KH2PO4 (0.5 eq, 8.65 mL, 17.32 mmol) was added in a single portion, followed by the addition of dithiothreitol (1.5 eq, 8.013 g, 51.95 mmol). A Vigreaux column was attached, and the solution was heated to 60°C for 2 h. The solvent was concentrated, and water was added to the mixture. The resulting wet solid was washed with deionized water and then transferred to a RBF using methanol (450 mL) and added 3-(chloromethyl)pyridine hydrochloride (1 eq, 5.6803 g, 34.63 mmol) and the resulting suspension was degassed by bubbling argon directly into the solution. This process used 3 consecutive balloons full of argon. Then, K2CO3 (3 eq, 14.359 g, 103.89 mmol) was added and the resulting reaction was stirred overnight at r.t. under an Ar atmosphere. The resulting suspension was concentrated to dryness to then be resuspended in Ethanol (300 mL) and a 1.0 M solution of NaOH (3.0 equiv., mL, mmol) was added and a Vigreaux column was attached to the flask and the suspension was stirred overnight at 85°C. The reaction was concentrated to dryness to give a yellow-brown solid. Methanol (300 ml) was added to the solid and the suspension stirred for 30 min to extract the product. The solid was filtered out and the washed with 200 ml of MeOH. The solution was concentrated, and the resulting crude was dry loaded in silica to purify in FCC. The column was run with a gradient going from 15% to 70% MeCN in EtOAc. The fractions containing product were combined to give 5-((pyridin-3-ylmethyl)thio)thiazol-2-amine (3.56 g, 15.93 mmol, 46% yield) as a yellow solid. ^{1}H-NMR (400 MHz, DMSO-d6) δ ppm 3.87 (s, 2 H) 6.73 (s, 1 H) 7.23 (s, 2 H) 7.33 (br. s., 1 H) 7.58 (s, 1 H) 8.32 (s, 1 H) 8.44 (d, J=4.75 Hz, 1 H). LRMS (ESI) m/z $[M+H]^+$ calcd for $C_9H_{10}N_3S_2$ 224.0, found 224.1

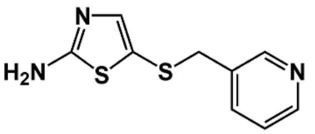

Synthesis of amino acid V

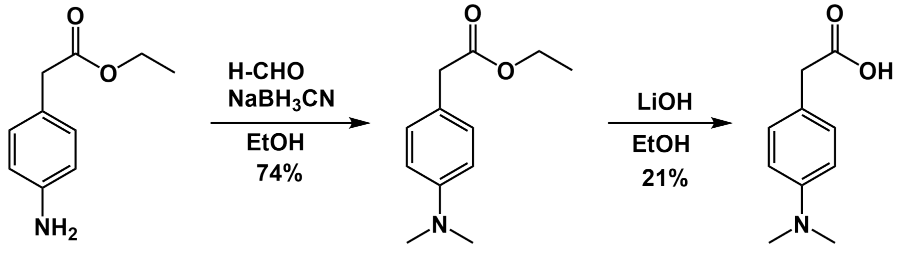

III IV V

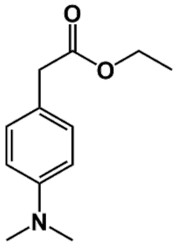

Ethyl 2-(4-(dimethylamino)phenyl)acetate (**IV**). Commercially available 4-aminophenylacetic acid ethyl ester (**III**) (6.00 g, 33.5 mmol) was suspended in EtOH (250 mL) and argon was bubbled into the solution for 15 min. Formaldehyde (7.48 mL, 100 mmol) was added followed by $NaBH_3CN$ (8.15 g, 134 mmol) and stir the reaction overnight under a Ar atmosphere. The reaction was quenched with HCl and any remaining acid was neutralized with $NaHCO_3$. The solution was concentrated, and the products were extracted with EtOAc (3 x 150 ml). The combined organics were washed with brine, then dried over Na2SO4, filtered and concentrated to give a crude that was purified in FCC using a gradient of EtOAc in Hexanes going from 20% to 60%. The fractions containing product were collected to give ethyl 2-(4-(dimethylamino)phenyl)acetate (**IV**) (5.2 g, 25.13 mmol, 75% yield) as a yellow viscous oil. ^{1}H-NMR (400 MHz, $CDCl_3$) δ ppm 1.26 (t, J=7.13 Hz, 3 H) 2.94 (s, 6 H) 3.52 (s, 2 H) 4.15 (q, J=7.13 Hz, 2 H) 6.65 - 6.76 (m, 2 H) 7.17 (d, J=8.63 Hz, 2 H). LRMS (ESI) m/z $[M+H]^+$ calcd for $C_{12}H_{18}NO_2$ 208.1, found 208.3

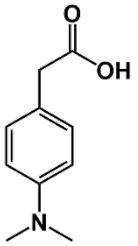

2-(4-(dimethylamino)phenyl)acetic acid (**V**). Dissolve ethyl 2-(4-(dimethylamino)phenyl)acetate (IV) (4.60 g, 23.8 mmol, 1.0 equiv.) in MeOH (200 mL) and add LiOH (2.28 g, 95.2 mmol, 4.0 equiv.) dissolved in water (15 mL) and stir overnight at r.t. Upon completion of the reaction, the excess base was quenched with conc. HCl, then concentrated and dry loaded into silica and then purified in FCC using a gradient from 10% to 50% acetone in Toluene with 1% AcOH as an additive. The fractions containing product were combined and concentrated to give 2-(4-(dimethylamino)phenyl)acetic acid (**V**) (1.83 g, 10.23 mmol, 43% yield). ^{1}H NMR (400 MHz, DMSO-d_6) δ ppm 2.85 (s, 6 H) 3.40 (s, 2 H) 6.63 - 6.69 (m, 2 H) 7.05 (d, J=8.63 Hz, 2 H) 12.13 (br. s., 1 H). LRMS (ESI) m/z $[M+H]^+$ calcd for $C_{10}H_{14}NO_2$ 180.1, found 180.2

Synthesis of carboxylic acid IX

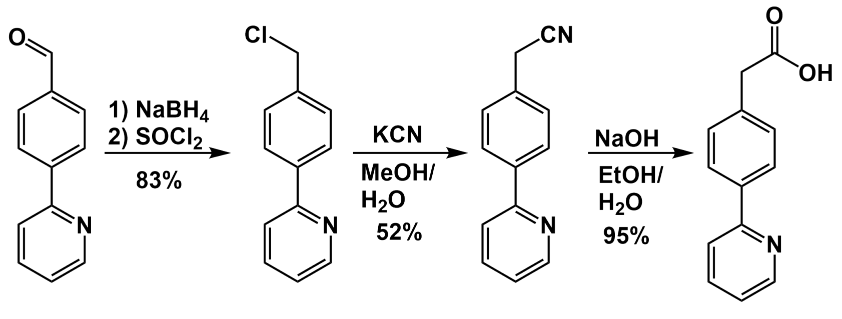

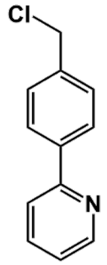

2-(4-(chloromethyl)phenyl)pyridine (**VII**). Step 1: 4-(2-pyridyl)benzaldehyde (**VI**) (600 mg, 3.28 mmol) dissolved in a mixture of EtOH (25 mL) and MeOH (10.1 mL) and an aqueous solution of NaBH4 (310 mg, 8.19 mmol, 10 mL of H_2O). The mixture was then sonicated until it was homogenous and then stirred overnight. The solution was concentrated to dryness and the product extracted in EtOAc (75 ml), washed with a saturated solution of $NaHCO_3$, water and brine (40 ml each). The organic phase was dried over $MgSO_4$ and concentrated to give (4-(pyridin-2-yl)phenyl)methanol (585 mg, 3.28 mmol, 96% yield) as colorless oil and was used as is in the next step. Step 2: (4-(pyridin-2-yl)phenyl)methanol (510 mg, 2.75 mmol) was dissolved in anhydrous DCM (12 mL), and thionyl chloride (1.20 mL, 16.5 mmol) was added dropwise at r.t. An off-white suspension was formed and was stirred overnight at r.t. The reaction was concentrated to dryness, and the product extracted in EtOAc (50ml), washed with a saturated solution of $NaHCO_3$ and brine. The organic phase was dried over MgSO4 and concentrated to give 2-(4-(chloromethyl)phenyl)pyridine (**VII**) (485 mg, 2.75 mmol, 86% yield, 83% yield over two steps) as coloress oil. ^{1}H NMR (400 MHz, $CDCl_3$) δ ppm 4.66 (s, 2 H) 7.25 (td, J=4.66, 2.44 Hz, 1 H) 7.42 - 7.60 (m, 2 H) 7.68 - 7.84 (m, 2 H) 7.95 - 8.06 (m, 2 H) 8.71 (dt, J=4.72, 1.33 Hz, 1 H). LRMS (ESI) m/z $[M+H]^+$ calcd for $C_{12}H_{11}ClN$ 204.1, found 204.1.

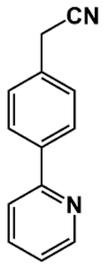

2-(4-(pyridin-2-yl)phenyl)acetonitrile (**VIII**). 2-(4-(chloromethyl)phenyl)pyridine (**VII**) (475 mg, 2.33 mmol) was dissolved in a mixture of EtOH (10 mL) and water (3 mL) and stirred at r.t. until reaching a homogenous solution. Then, KCN (175 mg, 2.68 mmol) was added and the resulting solution was sonicated for a few seconds and stirred overnight at 65°C. The product was then extracted in EtOAc (55ml), washed with water and brine. The organic phase was dried over $MgSO_4$ and then concentrated. The resulting pale yellow solid was triturated with 1 /4 EtOAc /Hex (10ml). The solid was then rinsed with the same solvent, dried under high vacuum and isolated 2-(4-(pyridin-2-yl)phenyl)acetonitrile (**VIII**) (235 mg, 2.33 mmol, 52% yield) as pale brown solid. ^{1}H NMR (400 MHz, $CDCl_3$) δ ppm 3.83 (s, 2 H) 7.16 - 7.34 (m, 2 H) 7.46 (m, J=8.51 Hz, 2 H) 7.69 - 7.86 (m, 2 H) 7.94 - 8.09 (m, 2 H) 8.71 (dt, J=4.69, 1.28 Hz, 1 H). LRMS (ESI) m/z $[M+H]^+$ calcd for $C_{13}H_{11}N_2$ 195.1, found 195.2.

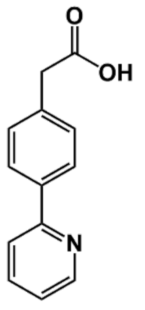

2-(4-(pyridin-2-yl)phenyl)acetic acid (**IX**). 2-(4-(pyridin-2-yl)phenyl)acetonitrile (VIII) (235 mg, 1.21 mmol) was dissolved in EtOH (6 mL) and an aqueous solution of NaOH (484 mg, 12.1 mmol, 1 ml) was added. The mixture was stirred overnight at 70°C. The mixture was cooled to r.t., evaporated the EtOH and the resulting solid was acidified with 1N HCl until pH ~ 3 and the product was extracted in EtOAc (55ml), washed with water and brine. The organic phase was dried over $MgSO_4$, filtered and concentrated to get 2-(4-(pyridin-2-yl)phenyl)acetic acid (**IX**) (245 mg, 1.21 mmol, 95% yield) as pale yellow oil. LRMS (ESI) m/z $[M+H]^+$ calcd for $C_{13}H_{12}NO_2$ 214.1 , found 214.2.

Synthesis of carboxylic acid XII

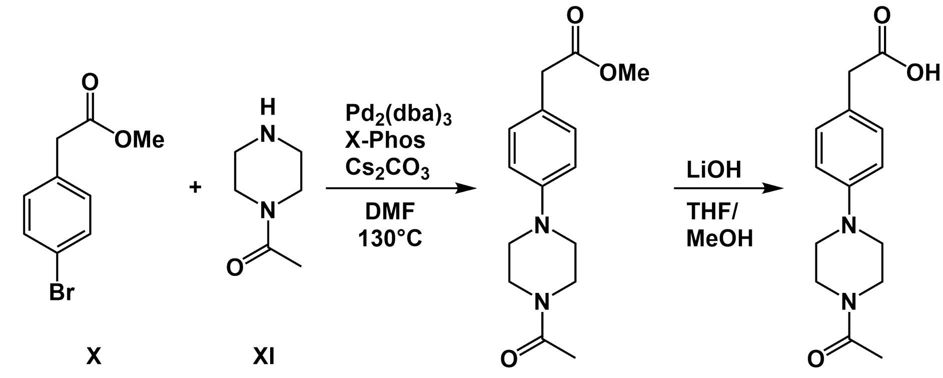

XII XIII

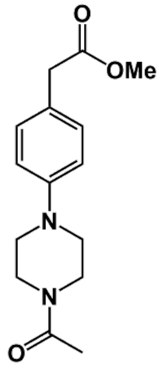

Methyl 2-(4-(4-acetylpiperazin-1-yl)phenyl)acetate (**XII**). 4-Bromophenylacetic acid methyl ester (**X**) (500 mg, 2.18 mmol) was dissolved in anhydrous DMF (6.00 mL) followed by the addition of Cs_2CO_3 (1.07 g, 3.27 mmol), $Pd_2(dba)_3$ (60.0 mg, 65.5 µmol) and X-Phos (41.6 mg, 87.3 µmol). The mixture was then degassed by bubbling nitrogen gas at r.t. for 10 min after which 1-acetylenepiperazine (**XI**) (560 mg, 4.37 mmol) was added. The reaction was heated at 130° C for 1 h after which the reaction turned black. The reaction was cooled down to r.t., and the product was extracted in EtOAc (55ml), washed with water and brine. The organic phase was dried over $MgSO_4$ and purified by Combi-flash (24 g column) using 5% EtOAc in DCM. The fractions containing product were combined, and concentrated to give methyl 2-(4-(4-acetylpiperazin-1-yl)phenyl)acetate (**XII**) (270 mg, 2.18 mmol, 45% yield) as a dark yellow oil. LRMS (ESI) m/z $[M+H]^+$ calcd for $C_{15}H_{21}N_2O_3$ 277.2, found 277.1.

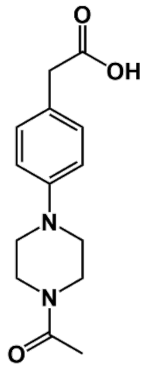

2-(4-(4-acetylpiperazin-1-yl)phenyl)acetic acid (**XIII**). Methyl 2-(4-(4-acetylpiperazin-1-yl)phenyl)acetate (**XII**) (270 mg, 977 µmol) was dissolved in a mixture of THF (16.0 mL), MeOH (3.00 mL), and water (2.0 mL) at r.t. A 4 N solution of LiOH (1.46 mL, 5.86 mmol) was added in a single portion and the solution was stirred overnight at 45°C. The solution was acidified with formic acid, concentrated and used as is in the next step. LRMS (ESI) m/z $[M+H]^+$ calcd for $C_{14}H_{19}N_2O_3$ 263.1, found 263.1.

Synthesis of aniline XV

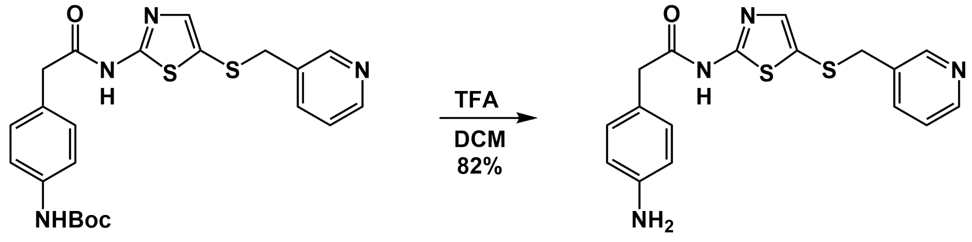

UOM-005100 XIV

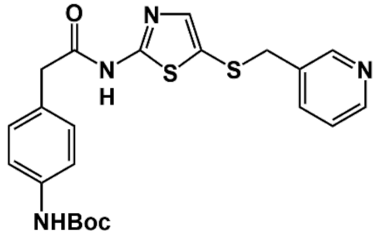

tert-butyl (4-(2-oxo-2-((5-((pyridin-3-ylmethyl)thio)thiazol-2-yl)amino)ethyl)phenyl)carbamate (**UOM-005100**). This compound was synthesized using the general method for peptide coupling (see methodology below). 2-[4-(tert-butoxycarbonylamino)phenyl]acetic acid (56.4 mg, 0.325 mmol, 1.3 equiv.), 5-((pyridin-3-ylmethyl)thio)thiazol-2-amine (**I**) (55.8 mg, 0.25 mmol, 1.0 equiv.), DIPEA (200 µL, 1.12 mmol, 4.5 equiv.), and BTFFH (118.6 mg, 0.38 mmol, 1.5 equiv.). The reaction was dry loaded in celite and was purified in C18 EZ Prep using a gradient from 30% to 95% MeOH in H_2O using 0.1% Formic acid as an additive. The fractions containing product were identified by LCMS and were combined, concentrated and freeze-dried to give **UOM-005100** (134 mg, 0.293 mmol, 26% yield). ^{1}H NMR (400 MHz, DMSO-d_6) δ ppm 1.46 (s, 9 H) 3.65 (s, 2 H) 3.98 (s, 2 H) 7.17 (d, J=8.61 Hz, 2 H) 7.25 (s, 1 H) 7.28 - 7.33 (m, 1 H) 7.38 (d, J=8.22 Hz, 2 H) 7.58 (d, J=7.83 Hz, 1 H) 8.29 (d, J=1.57 Hz, 1 H) 8.42 (dd, J=4.89, 1.76 Hz, 1 H) 9.29 (s, 1 H) 12.40 (br. s, 1 H). LRMS (ESI) m/z $[M+H]^+$ calcd for $C_{16}H_{15}N_4OS_2$ 343.1, found 343.1.

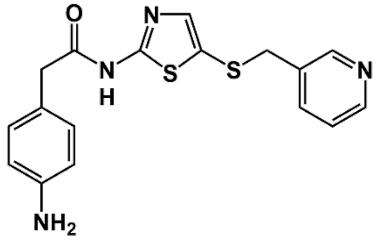

2-(4-aminophenyl)-N-(5-((pyridin-3-ylmethyl)thio)thiazol-2-yl)acetamide (**XIV**). Dissolve **UOM-005100** (500 mg, 1.10 mmol) in DCM (5.0 mL) and TFA (5.0 mL) and stir the mixture for 1 h. Then concentrate the mixture to dryness. Redissolve the mixture in MeOH and add IRA-67 resin until pH reaches ~ 8. Then filter the resin out and concentrate to give the crude compound, and purify it in FCC using 90% to 100% EtOAc in Hexanes. The fractions containing product were collected and concentrated to give **XIV** (320 mg, 0.90 mmol, 82 %) as a pale-yellow solid. ^{1}H NMR (400 MHz, DMSO-d_6) δ ppm 3.52 (s, 2 H) 3.99 (s, 2 H) 4.88 - 5.09 (m, 2 H) 6.50 (m, J=8.38 Hz, 2 H) 6.94 (d, J=8.50 Hz, 2 H) 7.24 (s, 1 H) 7.31 (dd, J=7.82, 4.82 Hz, 1 H) 7.58 (dt, J=7.88, 1.94 Hz, 1 H) 8.29 (d, J=1.88 Hz, 1 H) 8.42 (m, J=1.63 Hz, 1 H) 12.32 (s, 1 H). LRMS (ESI) m/z $[M+H]^+$ calcd for $C_{17}H_{17}N_4OS_2$ 357.1, found 357.1.

Synthesis of carboxylic acid XVII

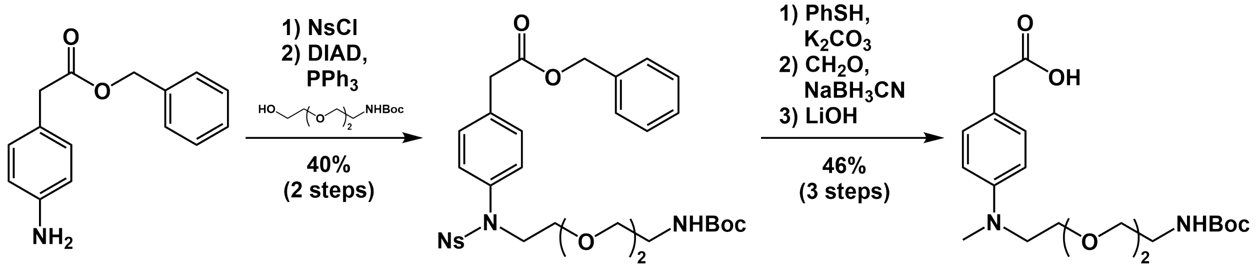

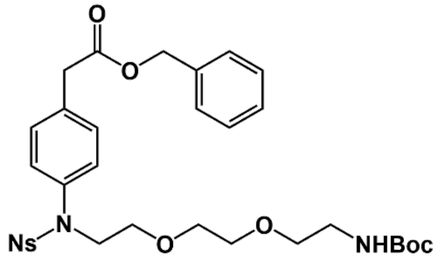

Benzyl 2-(4-((N-(2,2-dimethyl-4-oxo-3,8,11-trioxa-5-azatridecan-13-yl)-2-nitrophenyl)sulfonamido)phenyl)acetate (**XVI**). **Step 1:** **XV**(Currie *et al*, 2014) (2.67 g, 11.1 mmol, 1.0 equiv.) was dissolved in a mixture of DCM (60 mL) and pyridine (60 mL) followed by the addition of 2-nitrobenzenesulfonyl chloride (2.77 g, 12.2 mmol, 1.1 equiv.). The reaction was stirred overnight at r.t. under a nitrogen atmosphere. The reaction was dry loaded in silica and purified in FCC using a gradient of solvent going from 20 to 25% EtOAc in hexanes. The fractions containing product were combined to give benzyl 2-(4-((2-nitrophenyl)sulfonamido)phenyl)acetate (2.15 g, 45% yield). ^{1}H NMR (400 MHz, $CDCl_3$) δ ppm 3.61 (s, 2 H) 5.12 (s, 2 H) 7.12 - 7.25 (m, 5 H) 7.28 - 7.40 (m, 5 H) 7.53 - 7.59 (m, 1 H) 7.69 (td, J=7.75, 1.38 Hz, 1 H) 7.85 (ddd, J=11.29, 7.91, 1.19 Hz, 2 H). **Step 2:** The nosylated product (500 mg, 1.17 mmol, 1.0 equiv), triphenylphosphine (373 mg, 1.41 mmol, 1.2 equiv.), and tert-Butyl (2-(2-(2-hydroxyethoxy) ethoxy)ethyl)carbamate (310 uL, 1.29 mmol) were dissolved in DCM (11.0 mL). DIAD (261 mg, 1.29 mmol, 1.1 equiv.) was added in a single portion the mixture was stirred overnight at r.t. The mixture was then dry-loaded in silica and purified in FCC using a gradient from 35-45% EtOAc in Hexanes. The fractions containing product were combined and concentrated to give **XVI** (698 mg, 1.06 mmol, 90% yield) (40% yield over two steps). ^{1}H NMR (400 MHz, $CDCl_3$) δ ppm 1.38 - 1.53 (m, 9 H) 3.31 (q, J=4.96 Hz, 2 H) 3.52 (t, J=5.19 Hz, 2 H) 3.54 - 3.61 (m, 6 H) 3.68 (s, 2 H) 3.98 (t, J=5.88 Hz, 2 H) 5.02 (br. s., 1 H) 5.16 (s, 2 H) 7.19 - 7.27 (m, 4 H) 7.31 - 7.39 (m, 5 H) 7.40 - 7.46 (m, 1 H) 7.53 - 7.57 (m, 1 H) 7.63 (s, 2 H). LRMS (ESI) m/z $[M+H]^+$ calcd for $C_{32}H_{40}N_3O_{10}S$ 658.2, found 658.2.

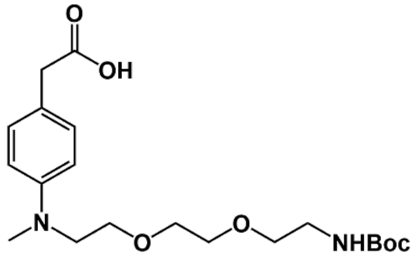

2-(4-((2,2-dimethyl-4-oxo-3,8,11-trioxa-5-azatridecan-13-yl)(methyl)amino)phenyl)acetic acid (**XVII**) **Step 1**: Dissolve **XVI** (698 mg, 1.06 mmol, 1.0 equiv.) in DMF (10.0 mL) and bubble the solution with Argon for 10 min. Then add 4-tert-butylthiophenol (220 µL, 1.27 mmol, 1.2 equiv.), K_2CO_3 (440 mg, 3.18 mmol) and close the vial and stir at r.t. for 4 h. The reaction was dry loaded in celite and purified in reverse phase using a Biotage C18 column with a gradient going from 60-95% MeOH in water with 0.1% formic acid. The fractions were left overnight, and the esters were esterified giving the corresponding methyl ester exclusively. The fractions containing product were combined and concentrated to

give methyl 2-(4-((2,2-dimethyl-4-oxo-3,8,11-trioxa-5-azatridecan-13-yl)amino)phenyl)acetate (314 mg, 75% yield). ^{1}H NMR (400 MHz, $CDCl_3$) δ ppm 1.45 (s, 9 H) 3.32 (m, J=5.29, 5.29, 5.29 Hz, 4 H) 3.52 (s, 2 H) 3.55 (t, J=5.19 Hz, 2 H) 3.63 (s, 4 H) 3.68 (s, 3 H) 3.70 (t, J=5.25 Hz, 2 H) 5.00 (br. s., 1 H) 6.60 (d, J=8.50 Hz, 2 H) 7.09 (d, J=8.50 Hz, 2 H). **Step 2**: The corresponding methyl ester intermediate (314 mg, 0.792 mmol, 1.0 equiv.) was dissolved in MeOH (8.00 mL) and then add formaldehyde (88.4 µL, 1.19 mmol, 1.5 equiv.) followed by NaBH3CN (99.5 mg, 1.58 mmol). The reaction was stir at r.t. overnight. Upon completion of the reaction, added AcOH (500 µL) to the reaction and stirred for 10 min followed by the addition of celite to dry load the compound. The crude was purified in the EZPrep using a 60 g Biotage Sfar C18 D column, using a solvent system going from 55% MeOH in H2O to 95% MeOH in water, with 0.1% formic acid as an additive. The fractions containing product were collected to give methyl 2-(4-((2,2-dimethyl-4-oxo-3,8,11-trioxa-5-azatridecan-13-yl)(methyl)amino)phenyl)acetate (303 mg, 93 %) as an amber oil. ^{1}H NMR (400 MHz, $CDCl_3$) δ ppm 1.45 (s, 9 H) 2.98 (s, 3 H) 3.32 (m, J=5.00 Hz, 2 H) 3.50 - 3.57 (m, 6 H) 3.59 (s, 4 H) 3.64 (t, J=6.00 Hz, 2 H) 3.68 (s, 3 H) 4.98 (br. s., 1 H) 6.68 (d, J=8.63 Hz, 2 H) 7.13 (d, J=8.63 Hz, 2 H). **Step 3**: Methyl 2-(4-((2,2-dimethyl-4-oxo-3,8,11-trioxa-5-azatridecan-13-yl)(methyl)amino)phenyl)acetate (303 mg, 738.0 umol) was dissolve in MeOH (6.00 mL) and LiOH (54.1 mg, 2.21 mmol) was added followed by 1 mL of water and the reaction was stir at 50°C for 2 h. Once the reaction had reached completion, the reaction was then concentrated to dryness and the mixture was then acidified with Acetic Acid Glacial (873 uL, 15.3 mmol), and the product was extracted from the solid with DCM (20 ml), and then EtOAc (20 ml x 2). The combined organics were combined and dried over Na_2SO_4 and concentrated to give **XVII** (192 mg, 0.484 mmol, 66 % yield) (46% over 3 steps) as a yellow solid. ^{1}H NMR (400 MHz, $CDCl_3$) δ ppm 1.44 (s, 9 H) 2.96 (s, 3 H) 3.22 (d, J=4.63 Hz, 2 H) 3.43 (t, J=4.50 Hz, 2 H) 3.47 - 3.59 (m, 8 H) 3.61 - 3.67 (m, 2 H) 4.91 - 5.04 (m, 1 H) 6.68 (d, J=8.63 Hz, 2 H) 7.13 (d, J=8.63 Hz, 2 H). LRMS (ESI) m/z $[M+H]^+$ calcd for $C_{20}H_{33}N_2O_6$ 397.2, found 397.3.

Synthesis of S656 derivatives

The following protocol was used as a general method for a peptide coupling(Due-Hansen *et al*, 2016), described as follows:

In a 8 mL vial, dissolve the corresponding carboxylic acid (1.0 - 1.5 equiv.) in DCM (0.6 - 0.8 M) and added DIPEA (4.0 - 6.0 equiv.) followed by BTFFH (1.5 equiv.). This mixture was stirred at r.t. for 30 min and then the corresponding amine (1.5 equiv.) was added, the vial was closed, and the reaction stirred at r.t. overnight. The reaction was dry loaded directly in silica and purified using the indicated gradient. The fractions containing product were collected and concentrated to give the desired product.

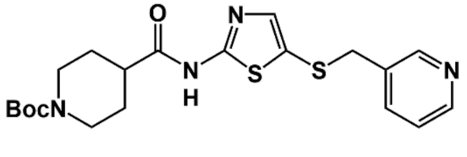

tert-butyl 4-((5-((pyridin-3-ylmethyl)thio)thiazol-2-yl) carbamoyl)piperidine-1-carboxylate (**UOM-005205**) This compound was synthesized using the general method for peptide coupling. N-Boc-DL-Isonipecotic acid (74.5 mg, 0.32 mmol, 1.3 equiv.), 5-(3-pyridylmethylsulfanyl)thiazol-2-amine (55.8 mg, 0.25 mmol, 1.0 equiv.), BTFFH (118.6 mg, 0.38 mmol, 1.5 equiv.), DIPEA (0.2 mL, 1.12 mmol, 4.5 equiv.). This crude was purified in C18 EZ Prep using a gradient from 30% to 95% MeOH in H_2O using 0.1%

formic acid as an additive. Obtained **UOM-005205** (85.3 mg, 0.196 mmol, 79% yield) as a yellow solid. ^{1}H NMR (400 MHz, DMSO-d_6) δ ppm 1.51 - 1.65 (m, 1 H) 1.79 (d, J=11.38 Hz, 2 H) 2.54 - 2.72 (m, 3 H) 3.11 (d, J=12.51 Hz, 4 H) 3.94 (s, 2 H) 7.19 (s, 1 H) 7.26 (dd, J=7.75, 4.75 Hz, 1 H) 7.53 - 7.58 (m, 1 H) 8.20 (br. s., 1 H) 8.22 (d, J=1.88 Hz, 1 H) 8.37 (dd, J=4.69, 1.44 Hz, 1 H). LRMS (ESI) m/z $[M+H]^+$ calcd for $C_{20}H_{27}N_4O_3S_2$ 435.2, found 435.1.

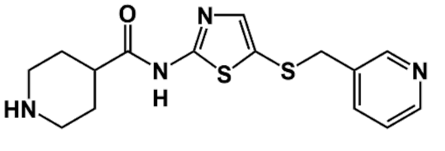

N-(5-((pyridin-3-ylmethyl)thio)thiazol-2-yl)piperidine-4-carboxamide (**UOM-005283**). Dissolve tert-butyl 4-((5-((pyridin-3-ylmethyl)thio)thiazol-2-yl) carbamoyl)piperidine-1-carboxylate (**UOM-005205**) (1 eq, 25 mg, 0.057 mmol) in DCM (0.5 mL) and add TFA (50 eq, 220 µL, 2.88 mmol) and stir the reaction for 30 min. Concentrate the reaction and redissolve it in DCM (5 mL) and add IRA-67 resin until the solution is basic. Filter the resin out and dry load the crude in celite. Purified the crude in the EZ Prep using a solvent system from 10 to 35% MeOH in H_2O. The fractions that contained product were combined to give **UOM-005283** (5.2 mg, 0.0155 mmol, 27 % yield) as a pale yellow solid. ^{1}H NMR (400 MHz, DMSO-d_6) δ ppm 1.40 (s, 9 H) 1.42 - 1.51 (m, 2 H) 1.72 - 1.82 (m, 2 H) 2.57 - 2.69 (m, 1 H) 2.71 - 2.84 (m, 2 H) 3.92 - 3.98 (m, 2 H) 4.00 (s, 2 H) 7.25 (s, 1 H) 7.33 (dd, J=7.82, 4.82 Hz, 1 H) 7.61 (dt, J=7.88, 1.94 Hz, 1 H) 8.29 (d, J=1.88 Hz, 1 H) 8.43 (dd, J=4.75, 1.50 Hz, 1 H) 12.22 - 12.29 (m, 1 H). LRMS (ESI) m/z $[M+H]^+$ calcd for $C_{15}H_{19}N_4OS_2$ 335.1, found 335.1.

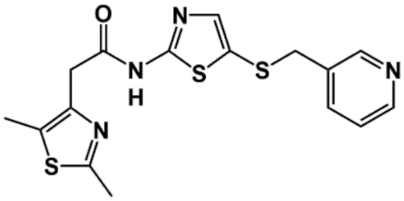

2-(2,5-dimethylthiazol-4-yl)-N-(5-((pyridin-3-ylmethyl)thio)thiazol-2-yl)acetamide (**UOM-005227**). This compound was synthesized using the general method for peptide coupling. 2-(2,5-Dimethyl-1,3-thiazol-4-yl)acetic acid (55.6 mg, 0.32 mmol, 1.3 equiv.), 5-(3-pyridylmethylsulfanyl)thiazol-2-amine (55.8 mg, 0.25 mmol, 1.0 equiv.), BTFFH (118.6 mg, 0.38 mmol, 1.5 equiv.), DIPEA (0.2 mL, 1.12 mmol, 4.5 equiv.). Purified in C18 EZ Prep using a gradient from 30% to 95% water in MeOH using 0.1% Formic acid as an additive. The fractions containing product were combined, concentrated, and freeze-dried to give **UOM-005227** (59.7 mg, 0.159 mmol, 63% yield) as a yellow solid. ^{1}H NMR (400 MHz, DMSO-d_6) δ ppm 2.33 (s, 3 H) 2.52 (s, 3 H) 3.79 (s, 2 H) 4.00 (s, 2 H) 7.27 (s, 1 H) 7.32 (ddd, J=7.82, 4.82, 0.75 Hz, 1 H) 7.60 (dt, J=7.91, 1.92 Hz, 1 H) 8.29 (d, J=1.88 Hz, 1 H) 8.42 (dd, J=4.75, 1.63 Hz, 1 H) 12.42 (s, 1 H). LRMS (ESI) m/z $[M+H]^+$ calcd for $C_{16}H_{17}N_4OS_3$ 377.1, found 377.1.

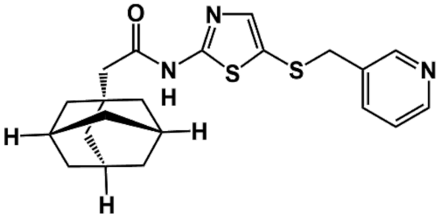

2-((3r,5r,7r)-adamantan-1-yl)-N-(5-((pyridin-3-ylmethyl)thio)thiazol-2-yl)acetamide (**UOM-005433**). This compound was synthesized using the general method for peptide coupling. 1-Adamantaneacetic acid (45.2 mg, 0.233 mmol, 1.3 equiv.), 5-((pyridin-3-ylmethyl)thio)thiazol-2-amine (**I**) (40 mg, 0.179 mmol, 1.0 equiv.), DIPEA (156 µL, 0.896 mmol, 5.0 equiv.), BTFFH (85.0 mg, 0.269 mmol, 1.5 equiv.). purified in the C18 prep column on the EZ Prep in a solvent system going from 30% to 95% MeOH in H_2O. The fractions containing product were identified and collected, then concentrated and lyopohilized to give **UOM-005433** (24.3 mg, 34 %) as a white solid. ^{1}H NMR (400 MHz, DMSO-d_6) δ ppm 1.57 (d, J=2.25 Hz, 9 H) 1.61 - 1.69 (m, 3 H) 1.92 (br. s., 3 H) 2.17 (s, 2 H) 4.01 (s, 2 H) 7.24 (s, 1 H) 7.32 (dd, J=7.75,

4.75 Hz, 1 H) 7.61 (dt, J=7.85, 1.89 Hz, 1 H) 8.30 (d, J=2.00 Hz, 1 H) 8.43 (dd, J=4.82, 1.56 Hz, 1 H) 12.09 (s, 1 H). LRMS (ESI) m/z $[M+H]^+$ calcd for $C_{21}H_{26}N_3OS_2$ 400.2, found 400.1.

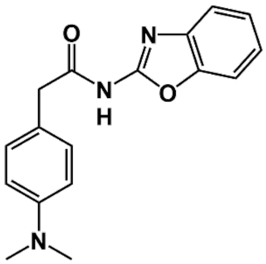

N-(benzo[d]oxazol-2-yl)-2-(4-(dimethylamino)phenyl)acetamide (UOM-005604). This compound was synthesized using the general method for peptide coupling. 2-(4-(dimethylamino)phenyl)acetic acid (**V**) (41.7 mg, 0.233 mmol, 1.0 equiv.), benzooxazol-2-ylamine (29.9 mg, 0.223 mmol, 1.0 equiv.), DIPEA (0.48 mL, 1.115 mmol, 5.0 equiv.), and BTFFH (110.5 mg, 0.35 mmol, 1.5 equiv.). Crude was dry loaded in silica and purified in regular phase using 10% to 20% Acetone in Toluene as the eluent. The fractions containing product were mixed and concentrated to give **UOM-005604** (7.70 mg, 0.027 mmol, 12% yield) as a white solid. ^{1}H NMR (400 MHz, DMSO-d_6) δ ppm 2.86 (s, 6 H) 3.66 (s, 2 H) 6.69 (d, J=8.51 Hz, 2 H) 7.14 (d, J=8.38 Hz, 2 H) 7.22 - 7.33 (m, 2 H) 7.58 (dd, J=13.32, 7.69 Hz, 2 H) 11.75 (br. s., 1 H). LRMS (ESI) m/z $[M+H]^+$ calcd for $C_{17}H_{18}N_3O_2$ 296.1, found 296.1.

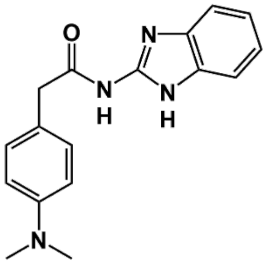

N-(1H-benzo[d]imidazol-2-yl)-2-(4-(dimethylamino)phenyl)acetamide (UOM-005603). This compound was synthesized using the general method for peptide coupling. 2-(4-(dimethylamino)phenyl)acetic acid (**V**) (41.7 mg, 0.233 mmol, 1.0 equiv.), 2-aminobenzimidazole (29.7 mg, 0.223 mmol, 1.0 equiv.), DIPEA (0.48 mL, 1.115 mmol, 5.0 equiv.), and BTFFH (110.5 mg, 0.35 mmol, 1.5 equiv.). Dry loaded the crude in celite and purified the compound in the EZ Prep, using a solvent system going from 15% to 40% MeOH in H_2O with 0.1% formic acid as an additive. The fractions containing product were collected and concentrated to give **UOM-005603** (24.5 mg, 0.060 mmol, 37% yield) as a white solid. ^{1}H NMR (400 MHz, DMSO-d_6) δ ppm 2.85 (s, 6 H) 3.61 (s, 2 H) 6.69 (d, J=8.63 Hz, 2 H) 7.00 - 7.11 (m, 2 H) 7.18 (d, J=8.50 Hz, 2 H) 7.36 - 7.48 (m, 2 H) 11.63 (br. s., 1 H) 11.98 (br. s., 1 H). LRMS (ESI) m/z $[M+H]^+$ calcd for $C_{17}H_{19}N_4O_2$ 295.2, found 295.1.

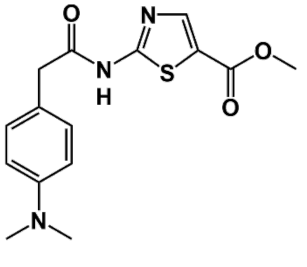

Methyl 2-(2-(4-(dimethylamino)phenyl)acetamido)thiazole-5-carboxylate (UOM-005602). This compound was synthesized using the general method for peptide coupling. 2-(4-(dimethylamino)phenyl)acetic acid (**V**) (41.7 mg, 0.233 mmol, 1.0 equiv.), methyl 2-aminothiazole-5-carboxylate (52.9 mg, 0.335 mmol, 1.5 equiv.), DIPEA (0.48 mL, 1.115 mmol, 5.0 equiv.), and BTFFH (110.5 mg, 0.35 mmol, 1.5 equiv.). The compound was dry loaded in celite and purified in the EZ Prep, using a solvent system going from 15% to 45% MeOH in H_2O with 0.1% formic acid as an additive. The fractions containing product were collected and concentrated to give **UOM-005602** (41.2 mg, 0.135 mmol, 58% yield). ^{1}H NMR (400 MHz, DMSO-d_6) δ ppm 2.85 (s, 6 H) 3.66 (s, 2 H) 3.79 (s, 3 H) 6.68 (d, J=8.50 Hz, 2 H) 7.13 (d, J=8.51 Hz, 2 H) 8.15 (s, 1 H) 12.72 (br. s., 1 H). LRMS (ESI) m/z $[M+H]^+$ calcd for $C_{15}H_{18}N_3O_3S$ 320.1, found 320.1.

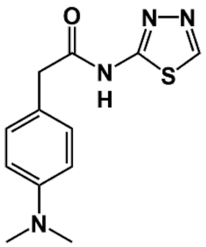

2-(4-(dimethylamino)phenyl)-N-(1,3,4-thiadiazol-2-yl)acetamide (**UOM-005601**). This compound was synthesized using the general method for peptide coupling. 2-(4-(dimethylamino)phenyl)acetic acid (**V**) (41.7 mg, 0.233 mmol, 1.0 equiv.), 2-amino-1,3,4-thiadiazole (33.8 mg, 0.335 mmol, 1.5 equiv.), DIPEA (0.48 mL, 1.115 mmol, 5.0 equiv.), and BTFFH (110.5 mg, 0.35 mmol, 1.5 equiv.). The compound was dry loaded in celite and purified in the EZ Prep, using a solvent system going from 10% to 45% MeOH in H_2O with 0.1% of formic acid as an additive. The fractions containing product were collected and concentrated to give **UOM-005601**. ^1H NMR (400 MHz, DMSO-d_6) δ ppm 2.85 (s, 6 H) 3.66 (s, 2 H) 6.68 (d, J=8.50 Hz, 2 H) 7.14 (d, J=8.50 Hz, 2 H) 9.14 (s, 1 H) 12.70 (s, 1 H). LRMS (ESI) m/z $[M+H]^+$ calcd for $C_{12}H_{15}N_4OS$ 263.1, found 263.1.

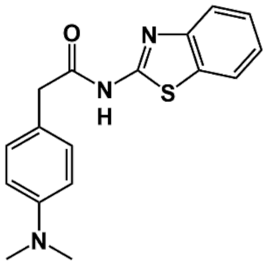

N-(benzo[d]thiazol-2-yl)-2-(4-(dimethylamino)phenyl)acetamide (**UOM-005600**). This compound was synthesized using the general method for peptide coupling. 2-(4-(dimethylamino)phenyl)acetic acid (**V**) (41.7 mg, 0.233 mmol, 1.0 equiv.), 2-aminobenzothiazole (50.2 mg, 0.335 mmol, 1.5 equiv.), DIPEA (0.48 mL, 1.115 mmol, 5.0 equiv.), and BTFFH (110.5 mg, 0.35 mmol, 1.5 equiv.). The compound was dry loaded in celite and purified in the EZ Prep, using a solvent system going from 20% to 85% MeOH in H2O with 0.1% formic acid as an additive. The fractions containing product were collected and concentrated to give **UOM-005600** (46.2 mg, 0.148 mmol, 67% yield) as a yellow solid. ^1H NMR (400 MHz, DMSO-d_6) δ ppm 2.77 - 2.93 (m, 6 H) 3.67 (s, 2 H) 6.69 (d, J=8.63 Hz, 2 H) 7.16 (d, J=8.50 Hz, 2 H) 7.25 - 7.33 (m, 1 H) 7.43 (t, J=7.63 Hz, 1 H) 7.73 (d, J=8.00 Hz, 1 H) 7.95 (d, J=7.88 Hz, 1 H) 12.49 (br. s., 1 H). LRMS (ESI) m/z $[M+H]^+$ calcd for $C_{17}H_{18}N_3OS$ 312.1, found 312.1.

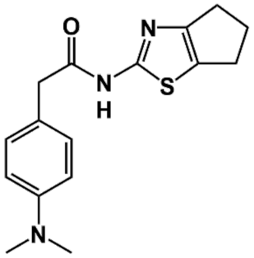

N-(5,6-dihydro-4H-cyclopenta[d]thiazol-2-yl)-2-(4-(dimethylamino)phenyl)acetamide (**UOM-005599**). This compound was synthesized using the general method for peptide coupling. 2-(4-(dimethylamino)phenyl)acetic acid (**V**) (41.7 mg, 0.233 mmol, 1.0 equiv.), 2-aminobenzothiazole (46.9 mg, 0.335 mmol, 1.5 equiv.), DIPEA (0.48 mL, 1.115 mmol, 5.0 equiv.), and BTFFH (110.5 mg, 0.35 mmol, 1.5 equiv.). The compound was dry loaded in celite and purified in the EZ Prep using a solvent system going from 15% to 70% MeOH in H_2O. The fractions containing product were collected and concentrated to give **UOM-005599** (39.5 mg, 0.131 mmol, 59% yield) as a dark yellow solid. ^1H NMR (400 MHz, DMSO-d_6) δ ppm 2.36 (quin, J=7.22 Hz, 2 H) 2.65 (t, J=7.25 Hz, 2 H) 2.79 (t, J=6.94 Hz, 2 H) 2.85 (s, 6 H) 3.56 (s, 2 H) 6.67 (d, J=8.63 Hz, 2 H) 7.12 (d, J=8.50 Hz, 2 H) 12.03 (br. s., 1 H). LRMS (ESI) m/z $[M+H]^+$ calcd for $C_{16}H_{20}N_3OS$ 302.1, found 302.1.

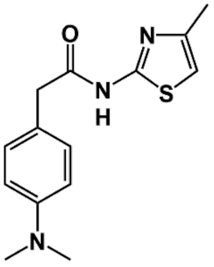

2-(4-(dimethylamino)phenyl)-N-(4-methylthiazol-2-yl)acetamide (**UOM-005598**). This compound was synthesized using the general method for peptide coupling. 2-(4-(dimethylamino)phenyl)acetic acid (**V**) (41.7 mg, 0.233 mmol, 1.0 equiv.), 2-amino-4-methylthiazole (38.2 mg, 0.335 mmol, 1.5 equiv.), DIPEA (0.48 mL, 1.115 mmol, 5.0 equiv.), and BTFFH (110.5 mg, 0.35 mmol, 1.5 equiv.). The compound was dry loaded in celite and purified in the EZ Prep using a solvent system going from 15% to 95% MeOH in water. The fractions containing product were collected and concentrated to give **UOM-005598** (29.4 mg, 0.107 mmol, 48% yield) as a light pink solid. ^{1}H NMR (400 MHz, DMSO-d_6) δ ppm 2.24 (s, 3 H) 2.85 (s, 6 H) 3.56 (s, 2 H) 6.67 (d, J=8.50 Hz, 2 H) 6.71 (s, 1 H) 7.12 (d, J=8.50 Hz, 2 H) 12.13 (s, 1 H). LRMS (ESI) m/z $[M+H]^+$ calcd for $C_{14}H_{18}N_3OS$ 276.1, found 276.1.

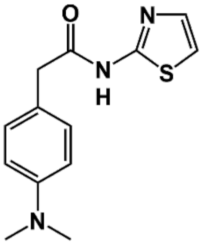

2-(4-(dimethylamino)phenyl)-N-(thiazol-2-yl)acetamide (**UOM-005597**). This compound was synthesized using the general method for peptide coupling. 2-(4-(dimethylamino)phenyl)acetic acid (**V**) (41.7 mg, 0.233 mmol, 1.0 equiv.), 2-aminothiazole (33.5 mg, 0.335 mmol, 1.5 equiv.), DIPEA (0.48 mL, 1.115 mmol, 5.0 equiv.), and BTFFH (110.5 mg, 0.35 mmol, 1.5 equiv.). The compound was dry loaded in celite and purified in the EZ Prep using a solvent system going from 15% to 95% MeOH in water. The fractions containing product were collected to give **UOM-005597** (32.9 mg, 0.126 mmol, 56% yield). ^{1}H NMR (400 MHz, DMSO-d_6) δ ppm 2.85 (s, 6 H) 3.60 (s, 2 H) 6.68 (d, J=8.63 Hz, 2 H) 7.14 (d, J=8.63 Hz, 2 H) 7.18 (d, J=3.63 Hz, 1 H) 7.45 (d, J=3.50 Hz, 1 H) 12.22 (s, 1 H). LRMS (ESI) m/z $[M+H]^+$ calcd for $C_{13}H_{16}N_3OS$ 262.1, found 262.1.

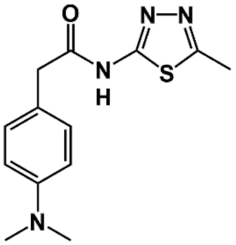

2-(4-(dimethylamino)phenyl)-N-(5-methyl-1,3,4-thiadiazol-2-yl)acetamide (**UOM-005596**). This compound was synthesized using the general method for peptide coupling. 2-(4-(dimethylamino)phenyl)acetic acid (**V**) (41.7 mg, 0.233 mmol, 1.0 equiv.), 2-aminothiazole (33.5 mg, 0.335 mmol, 1.5 equiv.), DIPEA (0.48 mL, 1.115 mmol, 5.0 equiv.), and BTFFH (110.5 mg, 0.35 mmol, 1.5 equiv.). The compound was dry loaded in celite and purified in the EZ Prep using a solvent system going from 15% to 95% MeOH in H_2O. The fractions containing product were collected to give **UOM-005596** (35.8 mg, 0.130 mmol, 58% yield) as an off white solid. ^{1}H NMR (400 MHz, DMSO-d_6) δ ppm 2.58 (s, 3 H) 2.85 (s, 6 H) 3.63 (s, 2 H) 6.67 (d, J=8.63 Hz, 2 H) 7.12 (d, J=8.63 Hz, 2 H) 12.50 (br. s., 1 H). LRMS (ESI) m/z $[M+H]^+$ calcd for $C_{13}H_{17}N_4OS$ 277.1, found 277.1.

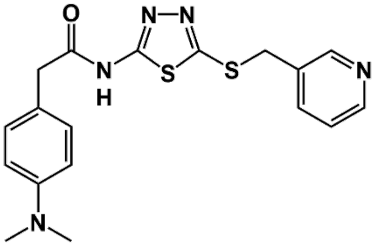

2-(4-(dimethylamino)phenyl)-N-(5-((pyridin-3-ylmethyl)thio)-1,3,4-thiadiazol-2-yl)acetamide (**UOM-005628**). This compound was synthesized using the general method for peptide coupling. 2-(4-(dimethylamino)phenyl)acetic acid (**V**) (32.1 mg, 0.179 mmol, 1.0 equiv.), 2-aminothiazole (33.5 mg, 0.335 mmol, 1.5 equiv.), DIPEA (0.48 mL, 1.115 mmol, 5.0 equiv.), and BTFFH (110.5 mg, 0.35 mmol, 1.5 equiv.). The compound was dry loaded in celite and purified in the EZ Prep using a gradient going from 15% to 60% MeOH in H2O. The fractions

containing product were combined to give **UOM-005628** (16.0 mg, 0.042 mmol, 23% yield). ^{1}H NMR (400 MHz, DMSO-d_6) δ ppm 2.85 (s, 6 H) 3.63 (s, 2 H) 4.48 (s, 2 H) 6.67 (d, J=8.63 Hz, 2 H) 7.11 (d, J=8.63 Hz, 2 H) 7.34 (dd, J=7.82, 4.82 Hz, 1 H) 7.80 (d, J=7.88 Hz, 1 H) 8.45 (d, J=4.50 Hz, 1 H) 8.56 (d, J=1.25 Hz, 1 H) 12.76 (br. s., 1 H). LRMS (ESI) m/z $[M+H]^+$ calcd for $C_{18}H_{20}N_5OS_2$ 386.1, found 386.1.

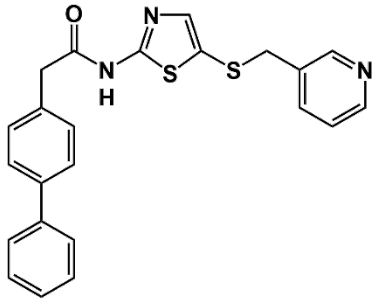

2-([1,1'-biphenyl]-4-yl)-N-(5-((pyridin-3-ylmethyl)thio)thiazol-2-yl)acetamide (**UOM-005429**). This compound was synthesized using the general method for peptide coupling. 4-biphenylacetic acid (49.4 mg, 0.233 mmol, 1.3 equiv.), 5-((pyridin-3-ylmethyl)thio)thiazol-2-amine (**I**) (40 mg, 0.179 mmol, 1.0 equiv.) DIPEA (156 µL, 0.896 mmol, 5.0 equiv.), and BTFFH (85 mg, 0.269 mmol, 1.5 equiv.). The reaction was then concentrated and redissolved in DMSO to get purified in the C18 prep column on the EZ Prep in a solvent system going from 30% to 95% MeOH in H_2O. The fractions containing product were identified and collected, then concentrated and lyophilized to give **UOM-005429** (32.6 mg, 0.078 mmol, 44% yield) as an off-white solid. ^{1}H NMR (400 MHz, DMSO-d_6) δ ppm 3.80 (s, 2 H) 3.99 (s, 2 H) 7.26 (s, 1 H) 7.31 (dd, J=7.75, 4.75 Hz, 1 H) 7.33 - 7.42 (m, 3 H) 7.46 (t, J=7.63 Hz, 2 H) 7.57 - 7.67 (m, 5 H) 8.28 (d, J=2.00 Hz, 1 H) 8.42 (dd, J=4.75, 1.50 Hz, 1 H) 12.49 (br. s., 1 H). LRMS (ESI) m/z $[M+H]^+$ calcd for $C_{23}H_{20}N_3OS_2$ 418.1, found 418.2.

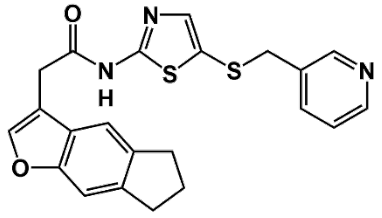

2-(6,7-dihydro-5H-indeno[5,6-b]furan-3-yl)-N-(5-((pyridin-3-ylmethyl)thio)thiazol-2-yl)acetamide (**UOM-005479**). This compound was synthesized using the general method for peptide coupling. 2-{5H,6H,7H-indeno[5,6-b]furan-3-yl}acetic Acid (50.6 mg, 0.233 mmol, 1.3 equiv.), 5-((pyridin-3-ylmethyl)thio)thiazol-2-amine (**I**) (40 mg, 0.179 mmol, 1.0 equiv.) DIPEA (156 µL, 0.896 mmol, 5.0 equiv.), and BTFFH (85 mg, 0.269 mmol, 1.5 equiv.). The reaction was then concentrated and redissolved in DMSO to get purified in the C18 prep column on the EZ Prep in a solvent system going from 30% to 95% MeOH in H_2O. The fractions containing product were identified and collected, then concentrated and lyophilized to give **UOM-005479** (4.90 mg, 0.012 mmol, 6.5% yield) as a white solid. ^{1}H NMR (400 MHz, DMSO-d_6) δ ppm 2.06 (quin, J=7.29 Hz, 2 H) 2.92 (q, J=7.67 Hz, 4 H) 3.82 (s, 2 H) 3.99 (s, 2 H) 7.26 (s, 1 H) 7.30 (dd, J=7.75, 4.75 Hz, 1 H) 7.39 (d, J=9.38 Hz, 2 H) 7.59 (d, J=7.88 Hz, 1 H) 7.78 (s, 1 H) 8.28 (d, J=1.75 Hz, 1 H) 8.41 (d, J=4.75 Hz, 1 H) 12.52 (br. s., 1 H). LRMS (ESI) m/z $[M+H]^+$ calcd for $C_{22}H_{20}N_3O_2S_2$ 422.1, found 422.2.

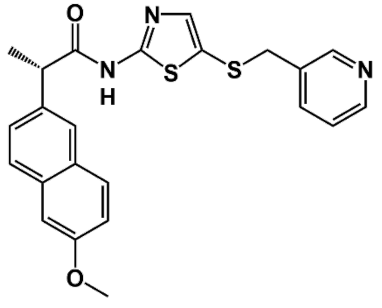

(S)-2-(6-methoxynaphthalen-2-yl)-N-(5-((pyridin-3-ylmethyl)thio)thiazol-2-yl)propenamide (**UOM-005431**). This compound was synthesized using the general method for peptide coupling. Naproxen (53.6 mg, 0.233 mmol, 1.3 equiv.), 5-((pyridin-3-ylmethyl)thio)thiazol-2-amine (**I**) (40 mg, 0.179 mmol, 1.0 equiv.), DIPEA (156 µL, 0.896 mmol, 5.0 equiv.), and BTFFH (85 mg, 0.269 mmol, 1.5 equiv.). The reaction was then concentrated and redissolved in DMSO to get purified in the C18 prep column on the

EZ Prep in a solvent system going from 30% to 95% MeOH in H_2O. The fractions containing product were identified and collected, then concentrated and lyophilized to give **UOM-005431** (59.5 mg, 0.129 mmol, 76%) as an off white solid. ^{1}H NMR (400 MHz, DMSO-d_6) δ ppm 1.51 (d, J=7.00 Hz, 3 H) 3.86 (s, 3 H) 3.98 (s, 2 H) 4.09 (q, J=6.92 Hz, 1 H) 7.15 (dd, J=8.94, 2.56 Hz, 1 H) 7.22 (s, 1 H) 7.26 - 7.33 (m, 2 H) 7.46 (dd, J=8.44, 1.69 Hz, 1 H) 7.58 (dt, J=7.88, 1.88 Hz, 1 H) 7.75 - 7.83 (m, 3 H) 8.28 (d, J=2.13 Hz, 1 H) 8.41 (dd, J=4.75, 1.63 Hz, 1 H) 12.46 (br. s., 1 H). LRMS (ESI) m/z $[M+H]^+$ calcd for $C_{23}H_{23}N_3O_2S_2$ 436.1, found 436.1.

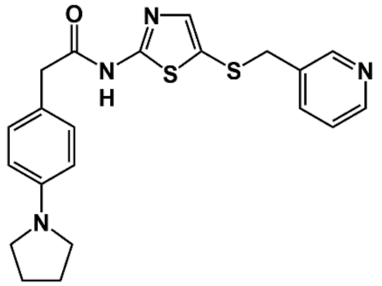

N-(5-((pyridin-3-ylmethyl)thio)thiazol-2-yl)-2-(4-(pyrrolidin-1-yl)phenyl)acetamide (**UOM-005608**). This compound was synthesized using the general method for peptide coupling. 2-(4-(pyrrolidin-1-yl)phenyl)acetic acid(Lang *et al*, 2014) (49.6 mg, 0.242 mmol, 1.2 equiv.), 5-((pyridin-3-ylmethyl)thio)thiazol-2-amine (**I**) (45 mg, 0.202 mmol, 1.0 equiv.), DIPEA (158 µL, 0.907 mmol, 4.5 equiv.), and BTFFH (95.6 mg, 0.302 mmol, 1.5 equiv.). Purify in Prep HPLC in a solvent system going from 30% to 95% MeOH in H_2O. The fractions containing product were identified and collected, then concentrated and lyophilized to give **UOM-005608** (45.0 mg, 0.110 mmol, 54% yield. ^{1}H NMR (400 MHz, DMSO-d_6) δ ppm 1.72 - 2.02 (m, 4 H) 3.18 (t, J=6.25 Hz, 4 H) 3.32 (br. s., 3 H) 3.57 (s, 2 H) 3.98 (s, 2 H) 6.48 (m, J=8.38 Hz,2 H) 7.10 (m, J=8.25 Hz, 2 H) 7.24 (s, 1 H) 7.31 (dd, J=7.69, 4.82 Hz, 1 H) 7.58 (d, J=7.75 Hz, 1 H) 8.28 (s, 1 H) 8.42 (d, J=4.75 Hz, 1 H) 12.34 (br. s., 1 H). LRMS (ESI) m/z $[M+H]^+$ calcd for $C_{21}H_{23}N_4OS_2$ 411.1, found 411.1.

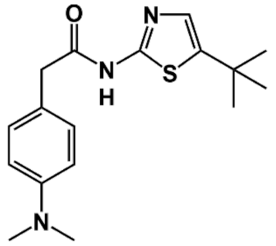

N-(5-(tert-butyl)thiazol-2-yl)-2-(4-(dimethylamino)phenyl)acetamide (**UOM-005605**). This compound was synthesized using the general method for peptide coupling. 2-(4-(dimethylamino)phenyl)acetic acid (**V**) (41.7 mg, 0.233 mmol, 1.0 equiv.), 5-tert-Butyl-1,3-thiazol-2-amine (34.8 mg, 0.233 mmol, 1.0 equiv.), DIPEA (0.48 mL, 1.115 mmol, 5.0 equiv.), and BTFFH (110.5 mg, 0.35 mmol, 1.5 equiv.). The compound was dry loaded in celite and purified in the EZ Prep using a solvent system going from 15% to 95% MeOH in H_2O. The fractions containing product were collected to give **UOM-005605** (38.4 mg, 0.121 mmol, 54% yield). ^{1}H NMR (400 MHz, DMSO-d_6) δ ppm 1.30 (s, 9 H) 2.85 (s, 6 H) 3.56 (s, 2 H) 6.67 (d, J=8.63 Hz, 2 H) 7.08 - 7.16 (m, 3 H) 12.03 (s, 1 H). LRMS (ESI) m/z $[M+H]^+$ calcd for $C_{17}H_{24}N_3OS$ 318.2, found 318.1.

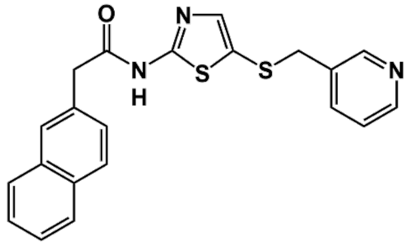

2-(naphthalen-2-yl)-N-(5-((pyridin-3-ylmethyl)thio)thiazol-2-yl)acetamide (**UOM-005428**). This compound was synthesized using the general method for peptide coupling. 2-Napthylacetic acid (43.4 mg, 0.233 mmol, 1.3 equiv.), 5-((pyridin-3-ylmethyl)thio)thiazol-2-amine (**I**) (40 mg, 0.179 mmol, 1.0 equiv.), DIPEA (156 µL, 0.896 mmol, 5.0 equiv.), and BTFFH (85 mg, 0.269 mmol, 1.5 equiv.). The reaction was then concentrated and redissolved in DMSO to get purified in the C18 prep column on the EZ Prep in

a solvent system going from 30% to 95% MeOH in H_2O. The fractions containing product were identified and collected, then concentrated and lyopohilized to give **UOM-005428** (32.7 mg, 0.084 mmol, 47%) as an off white solid. ^{1}H NMR (400 MHz, DMSO-d_6) δ ppm 3.93 (s, 2 H) 3.99 (s, 2 H) 7.26 (s, 1 H) 7.30 (dd, J=7.82, 4.82 Hz, 1 H) 7.44 - 7.54 (m, 3 H) 7.58 (dt, J=7.88, 1.88 Hz, 1 H) 7.82 (s, 1 H) 7.85 - 7.92 (m, 3 H) 8.28 (d, J=2.13 Hz, 1 H) 8.41 (dd, J=4.75, 1.50 Hz, 1 H) 12.51 (br. s., 1 H). LRMS (ESI) m/z $[M+H]^+$ calcd for $C_{21}H_{18}N_3OS_2$ 392.1, found 392.2.

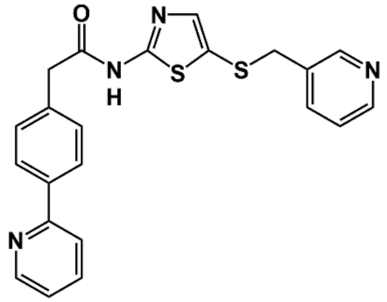

2-(4-(pyridin-2-yl)phenyl)-N-(5-((pyridin-3-ylmethyl)thio)thiazol-2-yl)acetamide (**UOM-005636**). This compound was synthesized using the general method for peptide coupling. 2-(4-(pyridin-2-yl)phenyl)acetic acid (**IX**) (74.5 mg, 0.349 mmol, 1.2 equiv.), 5-((pyridin-3-ylmethyl)thio)thiazol-2-amine (**I**) (65 mg, 0.291 mmol, 1.0 equiv.), DIPEA (228 µL, 1.31 mmol, 4.5 equiv.), and BTFFH (138 mg, 0.437 mmol, 1.5 equiv.). The reaction was then concentrated and redissolved in DMSO to get purified in the C18 prep column on the Prep HPLC in a solvent system going from 30% to 95% MeOH in H_2O. The fractions containing product were identified and collected, then concentrated and lyophilized to give **UOM-005636** (35.6 mg, 0.085 mmol, 29% yield) as a beige powder. ^{1}H NMR (400 MHz, DMSO-d_6) δ ppm 3.83 (s, 2 H) 4.00 (s, 2 H) 7.15 - 7.39 (m, 3 H) 7.43 (m, J=8.25 Hz, 2 H) 7.59 (dt, J=7.82, 1.72 Hz, 1 H) 7.80- 7.99 (m, 2 H) 8.05 (m, J=8.25 Hz, 2 H) 8.29 (br. s., 1 H) 8.42 (d, J=3.88 Hz, 1 H) 8.66 (d, J=4.25 Hz, 1 H) 12.51 (s, 1 H). LRMS (ESI) m/z $[M+H]^+$ calcd for $C_{22}H_{19}N_4OS_2$ 419.1, found 419.1.

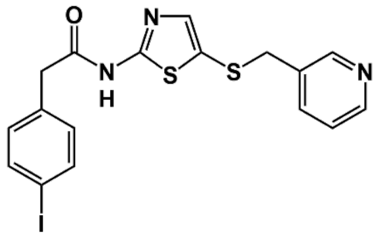

2-(4-iodophenyl)-N-(5-((pyridin-3-ylmethyl)thio)thiazol-2-yl)acetamide (**UOM-005200**). This compound was synthesized using the general method for peptide coupling. 2-(4-iodophenyl)acetic acid (85.1 mg, 0.325 mmol, 1.3 equiv.), 5-((pyridin-3-ylmethyl)thio)thiazol-2-amine (**I**) (55.8 mg, 0.25 mmol, 1.0 equiv.), DIPEA (200 µL, 1.12 mmol, 4.5 equiv.), and BTFFH (118.6 mg, 0.38 mmol, 1.5 equiv.). The reaction was dry loaded in celite and was purified in C18 EZ Prep using a gradient from 30% to 95% MeOH in H_2O using 0.1% Formic acid as an additive. The fractions containing product were identified by LCMS and were combined, concentrated and freeze-dried to give **UOM-005200** (71.4 mg, 0.153 mmol, 61% yield) as a yellow powder. ^{1}H NMR (400 MHz, DMSO-d_6) δ ppm 3.72 (s, 2 H) 3.99 (s, 2 H) 7.12 (d, J=8.25 Hz, 2 H) 7.26 (s, 1 H) 7.31 (dd, J=7.82, 4.82 Hz, 1 H) 7.59 (dt, J=7.85, 1.89 Hz, 1 H) 7.67 - 7.71 (m, 2 H) 8.28 (d, J=2.00 Hz, 1 H) 8.42 (dd, J=4.82, 1.56 Hz, 1 H) 12.46 (s, 1 H). LRMS (ESI) m/z $[M+H]^+$ calcd for $C_{17}H_{15}IN_3OS_2$ 468.0, found 467.9.

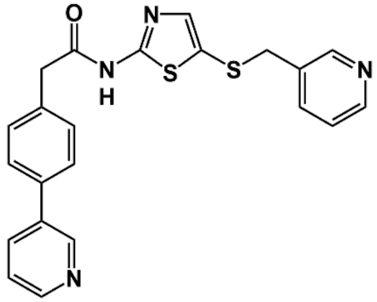

2-(4-(pyridin-3-yl)phenyl)-N-(5-((pyridin-3-ylmethyl)thio)thiazol-2-yl)acetamide (**UOM-005551**). 2-(4-iodophenyl)-N-(5-((pyridin-3-ylmethyl)thio)thiazol-2-yl)acetamide (**UOM-005200**) (1 eq, 45 mg, 0.0962 mmol) and bis(triphenylphosphine)palladium(II) chloride (0.05 eq, 3.3792 mg, 0.0048144 mmol) were suspended in dioxane (3 mL). Then pyridine-3-boroic acid (2.5 eq, 29.589 mg, 0.24072 mmol) was added, followed by aqueous solution of Na_2CO_3 (5 eq, 0.240 mL, 0.481 mmol). The mixture was stirred for 2 h at 100°C. The reaction was cooled down to r.t., added water (0.5ml) and EtOAc (5ml), sonicated a bit, filtered off the insoluble material over celite. Concentrated to nearly dryness, added DMSO (1.4ml) and 0.75uL of formic acid and directly purified by Prep-HPLC using a gradient from 30% to 95% MeOH in H_2O using 0.1% Formic acid as an additive. The fractions containing product were identified by LCMS and were combined, concentrated and freeze-dried to give **UOM-005551** (23.9 mg, 0.057 mmol, 59% yield) as an off white solid. ^{1}H NMR (400 MHz, DMSO-d_6) δ ppm 3.75 (s, 1 H) 3.82 (s, 11 H) 4.00 (s, 11 H) 7.23 - 7.35 (m, 12 H) 7.41 - 7.52 (m, 16 H) 7.59 (d, J=7.88 Hz, 6H) 7.69 (d, J=8.25 Hz, 11 H) 8.06 (d, J=8.00 Hz, 6 H) 8.29 (br. s., 5 H) 8.43 (br. s., 5 H) 8.57 (br. s., 5 H) 8.89 (br. s., 5 H) 12.51 (s, 6 H). LRMS (ESI) m/z $[M+H]^+$ calcd for $C_{22}H_{19}N_4OS_2$ 419.1, found 419.1.

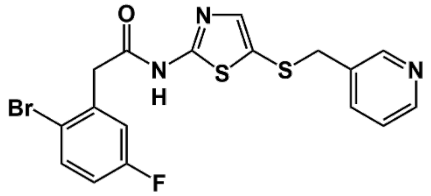

2-(2-bromo-5-fluorophenyl)-N-(5-((pyridin-3-ylmethyl)thio)thiazol-2-yl)acetamide (**UOM-005204**). This compound was synthesized using the general method for peptide coupling. 2-Bromo-5-fluorophenylacetic acid (75.7 mg, 0.325 mmol, 1.3 equiv.), 5-((pyridin-3-ylmethyl)thio)thiazol-2-amine (**I**) (55.8 mg, 0.25 mmol, 1.0 equiv.), DIPEA (200 µL, 1.12 mmol, 4.5 equiv.), and BTFFH (118.6 mg, 0.38 mmol, 1.5 equiv.). The reaction was dry loaded in celite and was purified in C18 EZ Prep using a gradient from 30% to 95% MeOH in H_2O using 0.1% Formic acid as an additive. The fractions containing product were identified by LCMS and were combined, concentrated and freeze-dried to give **UOM-005204**. ^{1}H NMR (400 MHz, DMSO-d_6) δ ppm 3.98 (s, 2 H) 4.00 (s, 2 H) 7.14 (td, J=8.60, 3.06 Hz, 1 H) 7.28 (s, 1 H) 7.29 - 7.37 (m, 2 H) 7.61 (dt, J=7.82, 1.91 Hz, 1 H) 7.65 (dd, J=8.82, 5.44 Hz, 1 H) 8.29 (d, J=2.00 Hz, 1 H) 8.42 (dd, J=4.75, 1.63 Hz, 1 H) 12.54 (br. s., 1 H). LRMS (ESI) m/z $[M+H]^+$ calcd for $C_{17}H_{14}BrFN_3OS_2$ 438.0, found 437.9.

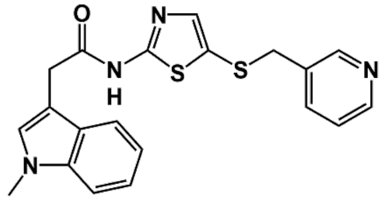

2-(1-methyl-1H-indol-3-yl)-N-(5-((pyridin-3-ylmethyl)thio)thiazol-2-yl)acetamide (**UOM-005228**). This compound was synthesized using the general method for peptide coupling. 2-(1-methylindol-3-yl)acetic acid (61.5 mg, 0.325 mmol, 1.3 equiv.), 5-((pyridin-3-ylmethyl)thio)thiazol-2-amine (**I**) (55.8 mg, 0.25 mmol, 1.0 equiv.), DIPEA (200 µL, 1.12 mmol, 4.5 equiv.), and BTFFH (118.6 mg, 0.38 mmol, 1.5 equiv.). The reaction was dry loaded in celite and was purified in C18 EZ Prep using a gradient from 30% to 95% MeOH in H_2O using 0.1% Formic acid as an additive. The fractions containing product were identified by LCMS and were combined, concentrated and freeze-dried to give **UOM-005228** (53.0 mg, 0.134 mmol, 54% yield). ^{1}H NMR

(400 MHz, DMSO-d_6) δ ppm 3.76 (s, 3 H) 3.84 (s, 2 H) 3.98 (s, 2 H) 7.03 (t, J=15.50 Hz, 1 H) 7.15 (t, J=7.57 Hz, 1 H) 7.25 (s, 2 H) 7.30 (dd, J=7.82, 4.82 Hz, 1 H) 7.40 (d, J=8.25 Hz, 1 H) 7.57 (d, J=7.88 Hz, 2 H) 8.28 (d, J=2.00 Hz, 1 H) 8.41 (dd, J=4.82, 1.56 Hz, 1 H) 12.31 - 12.51 (m, 1 H). LRMS (ESI) m/z $[M+H]^+$ calcd for $C_{20}H_{19}N_4OS_2$ 395.1, found 395.1.

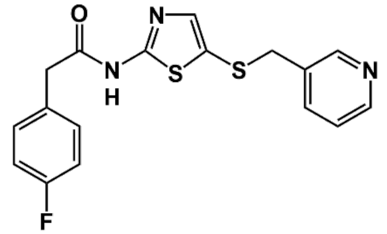

2-(4-fluorophenyl)-N-(5-((pyridin-3-ylmethyl)thio)thiazol-2-yl)acetamide (**UOM-005197**). This compound was synthesized using the general method for peptide coupling. 4-fluorophenylacetic acid (50.1 mg, 0.325 mmol, 1.3 equiv.), 5-((pyridin-3-ylmethyl)thio)thiazol-2-amine (**I**) (55.8 mg, 0.25 mmol, 1.0 equiv.), DIPEA (200 µL, 1.12 mmol, 4.5 equiv.), and BTFFH (118.6 mg, 0.38 mmol, 1.5 equiv.). The reaction was dry loaded in celite and was purified in C18 EZ Prep using a gradient from 30% to 95% MeOH in H_2O using 0.1% Formic acid as an additive. The fractions containing product were identified by LCMS and were combined, concentrated and freeze-dried to give **UOM-005197** (78.5 mg, 0.218 mmol, 87% yield). ^{1}H NMR (400 MHz, DMSO-d_6) δ ppm 3.75 (s, 2 H) 3.99 (s, 2 H) 7.15 (t, J=8.88 Hz, 2 H) 7.26 (s, 1 H) 7.28 - 7.40 (m, 3 H) 7.54 - 7.62 (m, 1 H) 8.28 (d, J=2.00 Hz, 1 H) 8.37 - 8.46 (m, 1 H) 12.42 - 12.49 (m, 1 H). LRMS (ESI) m/z $[M+H]^+$ calcd for $C_{17}H_{15}FN_3OS_2$ 360.1, found 360.0.

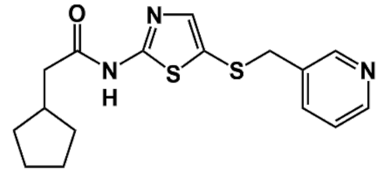

2-cyclopentyl-N-(5-((pyridin-3-ylmethyl)thio)thiazol-2-yl)acetamide (**UOM-005225**). This compound was synthesized using the general method for peptide coupling. Cyclopentylacetic acid (40.8 µL, 0.325 mmol, 1.3 equiv.), 5-((pyridin-3-ylmethyl)thio)thiazol-2-amine (**I**) (55.8 mg, 0.25 mmol, 1.0 equiv.), DIPEA (200 µL, 1.12 mmol, 4.5 equiv.), and BTFFH (118.6 mg, 0.38 mmol, 1.5 equiv.). The reaction was dry loaded in celite and was purified in C18 EZ Prep using a gradient from 30% to 95% MeOH in H_2O using 0.1% Formic acid as an additive. The fractions containing product were identified by LCMS and were combined, concentrated and freeze-dried to give **UOM-005225** (65.6 mg, 0.197 mmol, 79% yield). ^{1}H NMR (400 MHz, DMSO-d_6) δ ppm 1.08 - 1.20 (m, 2 H) 1.44 - 1.53 (m, 2 H) 1.53 - 1.64 (m, 2 H) 1.66 - 1.78 (m, 2 H) 2.19 (dquin, J=15.30, 7.65, 7.65, 7.65, 7.65 Hz, 1 H) 2.41 (d, J=7.50 Hz, 2 H) 4.01 (s, 2 H) 7.23 (s, 1 H) 7.32 (m, J=4.13 Hz, 1 H) 7.60 (dt, J=7.97, 1.89 Hz, 1 H) 8.29 (d, J=1.75 Hz, 1 H) 8.43 (dd, J=4.75, 1.63 Hz, 1 H) 11.98 - 12.27 (m, 1 H). LRMS (ESI) m/z $[M+H]^+$ calcd for $C_{16}H_{20}N_3OS_2$ 334.1, found 334.1.

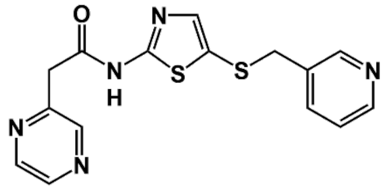

2-(pyrazin-2-yl)-N-(5-((pyridin-3-ylmethyl)thio)thiazol-2-yl)acetamide (**UOM-005482**). This compound was synthesized using the general method for peptide coupling. 2-Pyrazineacetic acid (44.9 mg, 0.325 mmol, 1.3 equiv.), 5-((pyridin-3-ylmethyl)thio)thiazol-2-amine (**I**) (55.8 mg, 0.25 mmol, 1.0 equiv.), DIPEA (200 µL, 1.12 mmol, 4.5 equiv.), and BTFFH (118.6 mg, 0.38 mmol, 1.5 equiv.). The reaction was dry loaded in celite and was purified in C18 EZ Prep using a gradient from 30% to 95% MeOH in H_2O using 0.1% Formic acid as an additive. The fractions containing product were identified by LCMS and were combined, concentrated and freeze-dried to give **UOM-005482** (17.6 mg, 0.051 mmol, 20% yield). ^{1}H NMR (400 MHz, DMSO-d_6) δ ppm 4.02 (s, 2 H) 4.06 (s, 2 H)

7.28 (s, 1 H) 7.36 (dd, J=7.75, 4.88 Hz, 1 H) 7.65 (dt, J=7.79, 1.92 Hz, 1 H) 8.31 (d, J=1.75 Hz, 1 H) 8.45 (dd, J=4.82, 1.56 Hz, 1 H) 8.55 - 8.57 (m, 1 H) 8.59 (dd, J=2.56, 1.56 Hz, 1 H) 8.66 (d, J=1.38 Hz, 1 H) 12.56 (s, 1 H). LRMS (ESI) m/z $[M+H]^+$ calcd for $C_{15}H_{14}N_5OS_2$ 344.1, found 344.0.

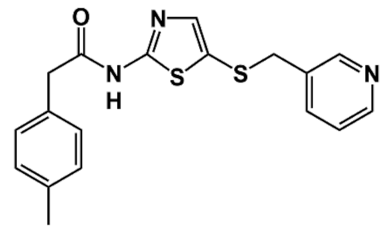

N-(5-((pyridin-3-ylmethyl)thio)thiazol-2-yl)-2-(p-tolyl)acetamide (**UOM-005202**). This compound was synthesized using the general method for peptide coupling. p-Tolylacetic acid (48.9 mg, 0.325 mmol, 1.3 equiv.), 5-((pyridin-3-ylmethyl)thio)thiazol-2-amine (**I**) (55.8 mg, 0.25 mmol, 1.0 equiv.), DIPEA (200 µL, 1.12 mmol, 4.5 equiv.), and BTFFH (118.6 mg, 0.38 mmol, 1.5 equiv.). The reaction was dry loaded in celite and was purified in C18 EZ Prep using a gradient from 30% to 95% MeOH in H_2O using 0.1% Formic acid as an additive. The fractions containing product were identified by LCMS and were combined, concentrated and freeze-dried to give **UOM-005202** (81.7 mg, 0.23 mmol, 92% yield). ^{1}H NMR (400 MHz, DMSO-d_6) δ ppm 2.27 (s, 3 H) 3.69 (s, 2 H) 3.99 (s, 2 H) 7.10 - 7.15 (m, 2 H) 7.16 - 7.21 (m, 2 H) 7.25 (s, 1 H) 7.31 (dd, J=7.57, 4.69 Hz, 1 H) 7.59 (dt, J=7.82, 1.97 Hz, 1 H) 8.28 (d, J=1.88 Hz, 1 H) 8.42 (dd, J=4.75, 1.63 Hz, 1 H) 12.42 (s, 1 H). LRMS (ESI) m/z $[M+H]^+$ calcd for $C_{18}H_{18}N_3OS_2$ 356.1, found 356.0.

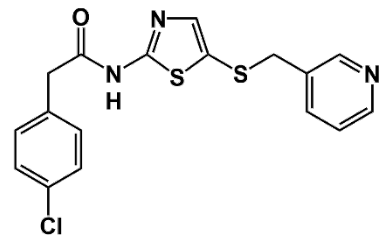

2-(4-chlorophenyl)-N-(5-((pyridin-3-ylmethyl)thio)thiazol-2-yl)acetamide (**UOM-005198**). This compound was synthesized using the general method for peptide coupling. p-Chlorophenylacetic acid (55.4 mg, 0.325 mmol, 1.3 equiv.), 5-((pyridin-3-ylmethyl)thio)thiazol-2-amine (**I**) (55.8 mg, 0.25 mmol, 1.0 equiv.), DIPEA (200 µL, 1.12 mmol, 4.5 equiv.), and BTFFH (118.6 mg, 0.38 mmol, 1.5 equiv.). The reaction was dry loaded in celite and was purified in C18 EZ Prep using a gradient from 30% to 95% MeOH in H_2O using 0.1% Formic acid as an additive. The fractions containing product were identified by LCMS and were combined, concentrated and freeze-dried to give **UOM-005198** (71.0 mg, 0.189 mmol, 76% yield) as a yellow powder. ^{1}H NMR (400 MHz, DMSO-d_6) δ ppm 3.77 (s, 2 H) 3.99 (s, 2 H) 7.26 (s, 1 H) 7.32 (s, 3 H) 7.36 - 7.41 (m, 2 H) 7.59 (dt, J=7.79, 1.92 Hz, 1 H) 8.28 (d, J=2.00 Hz, 1 H) 8.42 (dd, J=4.75, 1.63 Hz, 1 H) 12.47 (br. s., 1 H). LRMS (ESI) m/z $[M+H]^+$ calcd for $C_{17}H_{15}ClN_3OS_2$ 376.0, found 376.0.

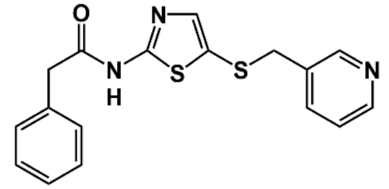

2-phenyl-N-(5-((pyridin-3-ylmethyl)thio)thiazol-2-yl)acetamide (**UOM-005190**). This compound was synthesized using the general method for peptide coupling. Phenylacetic acid (44.2 mg, 0.325 mmol, 1.3 equiv.), 5-((pyridin-3-ylmethyl)thio)thiazol-2-amine (**I**) (55.8 mg, 0.25 mmol, 1.0 equiv.), DIPEA (200 µL, 1.12 mmol, 4.5 equiv.), and BTFFH (118.6 mg, 0.38 mmol, 1.5 equiv.). The reaction was dry loaded in celite and was purified in C18 EZ Prep using a gradient from 30% to 95% MeOH in H_2O using 0.1% Formic acid as an additive. The fractions containing product were identified by LCMS and were combined, concentrated and freeze-dried to give **UOM-005190** (25.6 mg, 0.075 mmol, 30% yield) as a yellow solid. ^{1}H NMR (400 MHz, DMSO-d_6) δ ppm 3.75 (s, 2 H) 3.99 (s, 2 H) 7.23 - 7.36 (m, 7

H) 7.60 (d, J=7.63 Hz, 1 H) 8.29 (br. s., 1 H) 8.42 (d, J=3.13 Hz, 1 H) 12.46 (s, 1 H). LRMS (ESI) m/z $[M+H]^+$ calcd for $C_{17}H_{16}N_3OS_2$ 342.1, found 342.1.

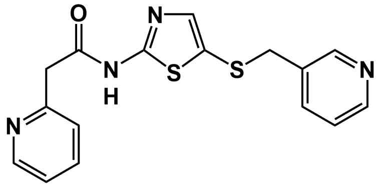

2-(pyridin-2-yl)-N-(5-((pyridin-3-ylmethyl)thio)thiazol-2-yl)acetamide (**UOM-005566**). This compound was synthesized using the general method for peptide coupling. 2-(2-Pyridyl)acetic acid (44.2 mg, 0.325 mmol, 1.3 equiv.), 5-((pyridin-3-ylmethyl)thio)thiazol-2-amine (**I**) (55.8 mg, 0.25 mmol, 1.0 equiv.), DIPEA (200 µL, 1.12 mmol, 4.5 equiv.), and BTFFH (118.6 mg, 0.38 mmol, 1.5 equiv.). The reaction was dry loaded in celite and was purified in C18 EZ Prep using a gradient from 30% to 95% MeOH in H_2O using 0.1% Formic acid as an additive. The fractions containing product were identified by LCMS and were combined, concentrated and freeze-dried to give **UOM-005566** (17 mg, 0.50 mmol, 20% yield). ^1H NMR (400 MHz, DMSO-d_6) δ ppm 3.96 (s, 2 H) 4.00 (s, 2 H) 7.27 (s, 1 H) 7.30 (s, 2 H) 7.38 (d, J=7.88 Hz, 1 H) 7.59 (dt, J=7.79, 1.92 Hz, 1 H) 7.77 (td, J=7.66, 1.81 Hz, 1 H) 8.29 (d, J=2.00 Hz, 1 H) 8.42 (dd, J=4.82, 1.56 Hz, 1 H) 8.49 (dd, J=4.82, 0.81 Hz, 1 H) 12.50 (br. s., 1 H). LRMS (ESI) m/z $[M+H]^+$ calcd for $C_{16}H_{15}N_4OS_2$ 343.1, found 343.1.

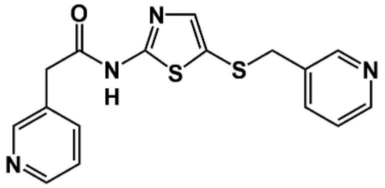

2-(pyridin-3-yl)-N-(5-((pyridin-3-ylmethyl)thio)thiazol-2-yl)acetamide (**UOM-005249**). This compound was synthesized using the general method for peptide coupling. 2-(2-2-(3-pyridyl)acetic acid hydrochloride (56.4 mg, 0.325 mmol, 1.3 equiv.), 5-((pyridin-3-ylmethyl)thio)thiazol-2-amine (**I**) (55.8 mg, 0.25 mmol, 1.0 equiv.), DIPEA (200 µL, 1.12 mmol, 4.5 equiv.), and BTFFH (118.6 mg, 0.38 mmol, 1.5 equiv.). The reaction was dry loaded in celite and was purified in C18 EZ Prep using a gradient from 30% to 95% MeOH in H_2O using 0.1% Formic acid as an additive. The fractions containing product were identified by LCMS and were combined, concentrated and freeze-dried to give **UOM-005249**. ^1H NMR (400 MHz, DMSO-d_6) δ ppm 3.83 (s, 2 H) 4.00 (s, 2 H) 7.27 (s, 1 H) 7.31 (dd, J=7.38, 4.75 Hz, 1 H) 7.37 (dd, J=7.44, 4.82 Hz, 1 H) 7.59 (dt, J=7.82, 1.97 Hz, 1 H) 7.72 (dt, J=7.88, 1.88 Hz, 1 H) 8.28 (d, J=1.88 Hz, 1 H) 8.42 (dd, J=4.75, 1.50 Hz, 1 H) 8.48 (dd, J=4.75, 1.50 Hz, 1 H) 8.50 (d, J=1.75 Hz, 1 H) 12.52 (s, 1 H). LRMS (ESI) m/z $[M+H]^+$ calcd for $C_{16}H_{15}N_4OS_2$ 343.1, found 343.1.

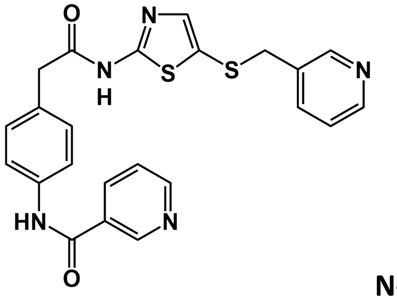

N-(4-(2-oxo-2-((5-((pyridin-3-ylmethyl)thio)thiazol-2-yl)amino)ethyl)phenyl)nicotinamide (**UOM-005475**). **XIV** (30.0 mg, 0.0842 mmol) was dissolved in DCM (1.00 mL) and pyridine (200 uL). Nicotinoyl chloride hydrochloride (44.9 mg, 0.252 mmol)

was added and the reaction was stirred overnight at r.t. The reaction was concentrated and redissolved in DMSO and purified in the EZ Prep with a C18 column using a solvent system from 35% to 95% MeOH in H_2O. The fractions containing product were combined and concentrated, then lyophilized to give **UOM-005475** (24.3 mg, 63 %) as a white solid. ^{1}H NMR (400 MHz, DMSO-d6) δ ppm 3.74 (s, 2 H) 4.00 (s, 2 H) 7.26 (s, 1 H) 7.28 - 7.34 (m, 3 H) 7.53 - 7.62 (m, 2 H) 7.72 (d, J=8.38 Hz, 2 H) 8.26 - 8.32 (m, 2 H) 8.42 (d, J=4.25 Hz, 1 H) 8.76 (d, J=4.50 Hz, 1 H) 9.10 (d, J=2.13 Hz, 1 H) 10.43 (s, 1 H) 12.46 (br. s., 1 H). LRMS (ESI) m/z $[M+H]^+$ calcd for $C_{23}H_{20}N_5O_2S_2$ 462.1, found 462.2.

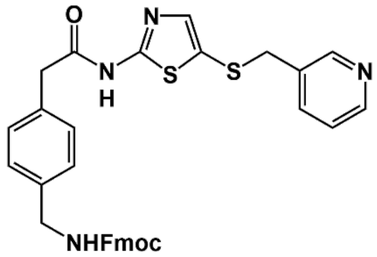

(9H-fluoren-9-yl)methyl (4-(2-oxo-2-((5-((pyridin-3-ylmethyl)thio)thiazol-2-yl)amino)ethyl)benzyl)carbamate (**UOM-005432**). This compound was synthesized using the general method for peptide coupling. Fmoc-4-aminomethyl-phenylacetic acid (90.2 mg, 0.233 mmol, 1.3 equiv.), 5-((pyridin-3-ylmethyl)thio)thiazol-2-amine (**I**) (40.0 mg, 0.179 mmol, 1.0 equiv.), DIPEA (156 µL, 0.896 mmol, 5.0 equiv.), and BTFFH (85.0 mg, 0.269 mmol, 1.5 equiv.). The reaction was then concentrated and redissolved in DMSO to get purified in the C18 prep column on the EZ Prep in a solvent system going from 30% to 95% MeOH in H_2O. The fractions containing product were identified and collected, then concentrated and lyophilized to give **UOM-005432** (21.7 mg, 0.037 mmol, 20% yield). 1H NMR (400 MHz, DMSO-d6) δ ppm 3.72 (s, 2 H) 3.99 (s, 2 H) 4.15 (d, J=6.00 Hz, 2 H) 4.22 (m, J=13.63 Hz, 1 H) 4.34 (d, J=6.75 Hz, 2 H) 7.14 - 7.19 (m, 2 H) 7.21 - 7.27 (m, 3 H) 7.31 (br. s., 3 H) 7.41 (s, 2 H) 7.59 (dt, J=7.85, 1.83 Hz, 1 H) 7.69 (d, J=7.50 Hz, 2 H) 7.82 (t, J=6.00 Hz, 1 H) 7.89 (d, J=7.50 Hz, 2 H) 8.28 (d, J=1.88 Hz, 1 H) 8.40 - 8.44 (m, 1 H) 12.41 (br. s., 1 H). LRMS (ESI) m/z $[M+H]^+$ calcd for $C_{33}H_{29}N_4O_3S_2$ 593.2, found 593.1.

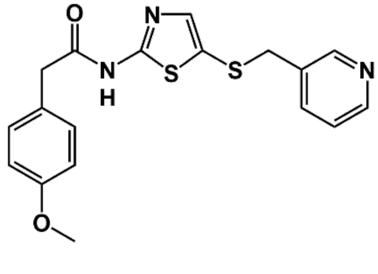

2-(4-methoxyphenyl)-N-(5-((pyridin-3-ylmethyl)thio)thiazol-2-yl)acetamide (**UOM-005201**). This compound was synthesized using the general method for peptide coupling. 4-Methoxyphenylacetic acid (54.1 mg, 0.325 mmol, 1.3 equiv.), 5-((pyridin-3-ylmethyl)thio)thiazol-2-amine (**I**) (55.8 mg, 0.25 mmol, 1.0 equiv.), DIPEA (200 µL, 1.12 mmol, 4.5 equiv.), and BTFFH (118.6 mg, 0.38 mmol, 1.5 equiv.). The reaction was dry loaded in celite and was purified in C18 EZ Prep using a gradient from 30% to 95% MeOH in H_2O using 0.1% Formic acid as an additive. The fractions containing product were identified by LCMS and were combined, concentrated and freeze-dried to give **UOM-005201** (81.4 mg, 0.22 mmol, 88% yield) as an off-white solid. ^{1}H NMR (400 MHz, DMSO-d_6) δ ppm 3.67 (s, 2 H) 3.73 (s, 3 H) 3.99 (s, 2 H) 6.88 (d, J=8.63 Hz, 2 H) 7.22 (d, J=8.63 Hz, 2 H) 7.25 (s, 1 H) 7.31 (dd, J=7.50, 5.00 Hz, 1 H) 7.59 (dt, J=7.79, 1.99 Hz, 1 H) 8.28 (d, J=1.88 Hz, 1 H) 8.42 (dd, J=4.75, 1.63 Hz, 1 H) 12.41 (br. s., 1 H). LRMS (ESI) m/z $[M+H]^+$ calcd for $C_{18}H_{18}N_3O_2S_2$ 372.1, found 372.1.

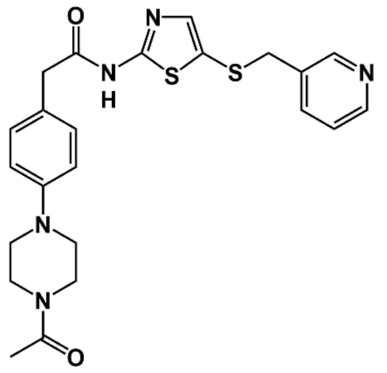

2-(4-(4-acetylpiperazin-1-yl)phenyl)-N-(5-((pyridin-3-ylmethyl)thio)thiazol-2-yl)acetamide (**UOM-005614**). This compound was synthesized using the general method for peptide coupling. **XIII** (80.3 mg, 0.306 mmol, 1.2 equiv.), 5-((pyridin-3-ylmethyl)thio)thiazol-2-amine (**I**) (57.0 mg, 0.255 mmol, 1.0 equiv.), DIPEA (200 µL, 1.12 mmol, 4.5 equiv.), and BTFFH (121 mg, 0.383 mmol, 1.5 equiv.). Concentrated the reaction and acidified the cruse with formic acid, followed by dissolving the crude in DMSO and purifying in the prep-HPLC with a solvent gradient going from 25% to 95% MeOH in water with 0.1% formic acid as an additive. The fractions containing product were identified by LCMS and were combined, concentrated and freeze-dried to give **UOM-005614** (41.5 mg, 0.090 mmol, 35% yield). ^{1}H NMR (400 MHz, DMSO-d_6) δ ppm 1.96 - 2.12 (m, 4 H) 2.97 - 3.18 (m, 4 H) 3.33 (s, 2 H) 3.52 - 3.61 (m, 4 H) 3.64 (s, 2 H) 4.00 (s, 2 H) 6.92 (m, J=8.75 Hz, 2 H) 7.18 (m, J=8.75 Hz, 2 H) 7.24 - 7.46 (m, 2 H) 7.59 (dt, J=7.85, 1.95 Hz, 1 H) 8.29 (d, J=1.88 Hz, 1 H) 8.43 (dd, J=4.75, 1.63 Hz, 1 H) 12.40 (br. s., 1 H). LRMS (ESI) m/z $[M+H]^+$ calcd for $C_{23}H_{26}N_5O_2S_2$ 468.2, found 468.1.

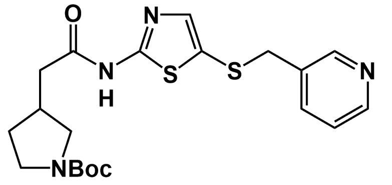

tert-butyl 3-(2-oxo-2-((5-((pyridin-3-ylmethyl)thio)thiazol-2-yl)amino)ethyl)pyrrolidine-1-carboxylate (**UOM-005226**). This compound was synthesized using the general method for peptide coupling. (R)-N-Boc-3-pyrrolidineacetic acid (74.5 mg, 0.325 mmol, 1.3 equiv.), 5-((pyridin-3-ylmethyl)thio)thiazol-2-amine (**I**) (55.8 mg, 0.25 mmol, 1.0 equiv.), DIPEA (200 µL, 1.12 mmol, 4.5 equiv.), and BTFFH (118.6 mg, 0.38 mmol, 1.5 equiv.). The reaction was dry loaded in celite and was purified in C18 EZ Prep using a gradient from 30% to 95% MeOH in H_2O using 0.1% Formic acid as an additive. The fractions containing product were identified by LCMS and were combined, concentrated and freeze-dried to give **UOM-005226**. ^{1}H NMR (400 MHz, DMSO-d_6) δ ppm 1.39 (s, 9 H) 1.44 - 1.60 (m, 1 H) 1.88 - 2.03 (m, 1 H) 2.51 - 2.54 (m, 2 H) 2.81 - 2.92 (m, 1 H) 3.10 - 3.23 (m, 1 H) 3.28 - 3.31 (m, 1 H) 3.38 - 3.50 (m, 1 H) 4.01 (s, 2 H) 7.24 (s, 1 H) 7.32 (dd, J=7.82, 4.82 Hz, 1 H) 7.60 (dt, J=7.88, 1.94 Hz, 1 H) 8.29 (d, J=1.75 Hz, 1 H) 8.43 (dd, J=4.75, 1.63 Hz, 1 H) 12.21 - 12.26 (m, 1 H). LRMS (ESI) m/z $[M+H]^+$ calcd for $C_{23}H_{26}N_5O_2S_2$ 435.1, found 435.1.

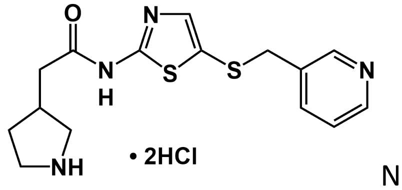

N-(5-((pyridin-3-ylmethyl)thio)thiazol-2-yl)-2-(pyrrolidin-3-yl)acetamide bis hydrochloride (**UOM-005300**). Dissolve **UOM-005226** (1 eq, 25 mg, 0.057 mmol) in DCM (0.5 mL) and add a 4.0 M solution of hydrogen chloride in Dioxane (50 eq, 0.72 mL, 2.87

mmol). Stir the mixture for 30 min and concentrate. Then lyophilize the sample to give **UOM-005300** (22 mg, 0.054 mmol, 94% yield) as a white powder. ^{1}H NMR (400 MHz, DMSO-d_6) δ ppm 1.55 (dd, J=12.69, 8.57 Hz, 1 H) 2.07 (dd, J=12.26, 4.88 Hz, 1 H) 2.53 - 2.61 (m, 1 H) 2.72 - 2.84 (m, 1 H) 3.02 - 3.14 (m, 1 H) 3.21 (dd, J=7.75, 4.00 Hz, 1 H) 3.28 - 3.37 (m, 1 H) 3.48 (dd, J=11.38, 4.38 Hz, 1 H) 3.65 - 3.73 (m, 1 H) 7.28 (s, 1 H) 7.99 (dd, J=7.88, 5.63 Hz, 1 H) 8.34 (d, J=8.00 Hz, 1 H) 8.69 (s, 1 H) 8.82 (d, J=5.50 Hz, 1 H) 9.32 (d, J=15.88 Hz, 2 H) 12.42 (br. s., 1 H). LRMS (ESI) m/z $[M+H]^+$ calcd for $C_{15}H_{18}N_4OS_2$ 335.1, found 335.1.

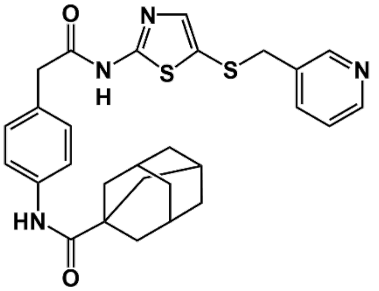

N-(4-(2-oxo-2-((5-((pyridin-3-ylmethyl)thio)thiazol-2-yl)amino)ethyl)phenyl)adamantane-1-carboxamide (**UOM-005477**). **XIV** (30.0 mg, 0.0842 mmol) was dissolved in DCM (1.00 mL) and pyridine (200 uL). 1-Adamantanecarbonyl chloride (50.2 mg, 0.252 mmol) was added and the reaction was stirred overnight at r.t. The reaction was concentrated and redissolved in DMSO and purified in the EZ Prep with a C18 column using a solvent system from 35% to 95% MeOH in H_2O. The fractions containing product were combined and concentrated, then lyophilized to give **UOM-005477** (20.5 mg, 0.040 mmol, 47% yield). ^{1}H NMR (400 MHz, DMSO-d_6) δ ppm 1.70 (br. s., 6 H) 1.89 (d, J=2.25 Hz, 6 H) 2.01 (br. s., 3 H) 3.68 (s, 2 H) 3.99 (s, 2 H) 7.20 (d, J=8.51 Hz, 2 H) 7.25 (s, 1 H) 7.31 (dd, J=7.75, 4.88 Hz, 1 H) 7.59 (d, J=8.50 Hz, 3 H) 8.29 (d, J=1.75 Hz, 1 H) 8.42 (d, J=4.63 Hz, 1 H) 9.09 (s, 1 H) 12.42 (br. s., 1 H). LRMS (ESI) m/z $[M+H]^+$ calcd for $C_{28}H_{31}N_4O_2S_2$ 519.2, found 519.2.

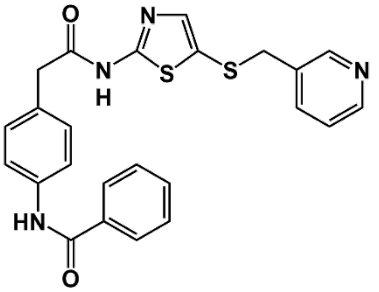

N-(4-(2-oxo-2-((5-((pyridin-3-ylmethyl)thio)thiazol-2-yl)amino)ethyl)phenyl)benzamide (**UOM-005478**). **XIV** (30.0 mg, 0.0842 mmol) was dissolved in DCM (1.00 mL) and pyridine (200 µL). Benzoyl chloride (9.8 µL, 0.0842 mmol) was added and the reaction was stirred overnight at r.t. The reaction was concentrated and redissolved in DMSO and purified in the EZ Prep with a C18 column using a solvent system from 35% to 95% MeOH in H_2O. The fractions containing product were combined and concentrated, then lyophilized to give **UOM-005478** (21.8 mg, 0.047 mmol, 56% yield) as a yellow powder. ^{1}H NMR (400 MHz, DMSO-d_6) δ ppm 3.73 (s, 2 H) 4.00 (s, 2 H) 7.23 - 7.35 (m, 4 H) 7.53 (m, J=7.75 Hz, 2 H) 7.59 (dd, J=7.13, 3.13 Hz, 2 H) 7.72 (d, J=8.38 Hz, 2 H) 7.95 (d, J=7.38 Hz, 2 H) 8.29 (d, J=1.88 Hz, 1 H) 8.42 (d, J=4.75 Hz, 1 H) 10.24 (s, 1 H) 12.45 (br. s., 1 H). LRMS (ESI) m/z $[M+H]^+$ calcd for $C_{24}H_{21}N_4O_2S_2$ 461.1, found 461.1.

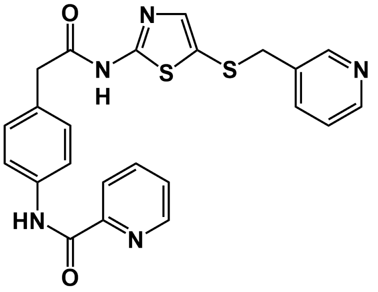

N-(4-(2-oxo-2-((5-((pyridin-3-ylmethyl)thio)thiazol-2-yl)amino)ethyl)phenyl)picolinamide (**UOM-005474**). **XIV** (30.0 mg, 0.0842 mmol) was dissolved

in DCM (1.00 mL) and pyridine (200 µL). Picolinoyl chloride hydrochloride (44.9 mg, 0.0842 mmol) was added and the reaction was stirred overnight at r.t. The reaction was concentrated and redissolved in DMSO and purified in the EZ Prep with a C18 column using a solvent system from 35% to 95% MeOH in H_2O. The fractions containing product were combined and concentrated, then lyophilized to give **UOM-005474** (27.9 mg, 0.060 mmol, 72% yield) as an off-white solid. ^{1}H NMR (400 MHz, DMSO-d_6) δ ppm 3.74 (s, 2 H) 3.99 (s, 2 H) 7.26 (s, 1 H) 7.27 - 7.34 (m, 3 H) 7.59 (d, J=7.75 Hz, 1 H) 7.68 (m, J=4.88 Hz, 1 H) 7.85 (d, J=8.38 Hz, 2 H) 8.07 (m, J=14.63 Hz, 1 H) 8.13 - 8.18 (m, 1 H) 8.29 (d, J=2.00 Hz, 1 H) 8.42 (d, J=4.63 Hz, 1 H) 8.74 (d, J=4.63 Hz, 1 H) 10.62 (s, 1 H) 12.46 (br. s., 1 H). LRMS (ESI) m/z $[M+H]^+$ calcd for $C_{23}H_{20}N_5O_2S_2$ 462.1, found 462.1.

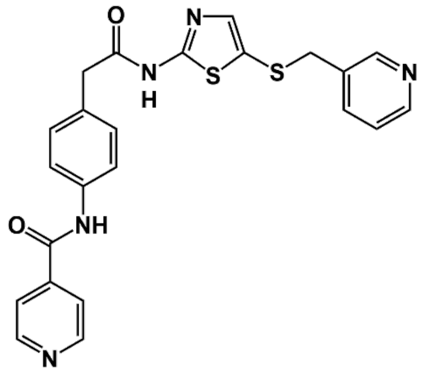

N-(4-(2-oxo-2-((5-((pyridin-3-ylmethyl)thio)thiazol-2-yl)amino)ethyl)phenyl)isonicotinamide (**UOM-005476**). **XIV** (30.0 mg, 0.0842 mmol) was dissolved in DCM (1.00 mL) and pyridine (200 µL). Isonicotinoyl chloride hydrochloride (44.9 mg, 0.0842 mmol) was added and the reaction was stirred overnight at r.t. The reaction was concentrated and redissolved in DMSO and purified in the EZ Prep with a C18 column using a solvent system from 35% to 95% MeOH in H_2O. The fractions containing product were combined and concentrated, then lyophilized to give **UOM-005476** (9.30 mg, 0.020 mmol, 24% yield) as a white solid. ^{1}H NMR (400 MHz, DMSO-d_6) δ ppm 3.74 (s, 2 H) 3.99 (s, 2 H) 7.26 (s, 1 H) 7.28 - 7.34 (m, 3 H) 7.59 (d, J=7.75 Hz, 1 H) 7.72 (d, J=8.25 Hz, 2 H) 7.86 (d, J=5.50 Hz, 2 H) 8.29 (d, J=1.63 Hz, 1 H) 8.42 (d, J=4.63 Hz, 1 H) 8.78 (d, J=5.38 Hz, 2 H) 10.49 (s, 1 H) 12.46 (br. s., 1 H). LRMS (ESI) m/z $[M+H]^+$ calcd for $C_{23}H_{20}N_5O_2S_2$ 462.1, found 462.1.

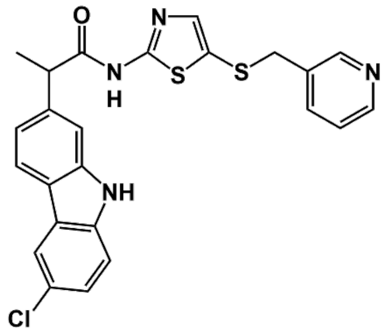

2-(6-chloro-9H-carbazol-2-yl)-N-(5-((pyridin-3-ylmethyl)thio)thiazol-2-yl)propenamide (**UOM-005447**). This compound was synthesized using the general method for peptide coupling. Carprofen (63.7 mg, 0.233 mmol, 1.3 equiv.), 5-((pyridin-3-ylmethyl)thio)thiazol-2-amine (**I**) (40.0 mg, 0.179 mmol, 1.0 equiv.), DIPEA (156 µL, 0.896 mmol, 5.0 equiv.), and BTFFH (85.0 mg, 0.269 mmol, 1.5 equiv.). The reaction was dry loaded in celite and was purified in C18 EZ Prep using a gradient from 30% to 95% MeOH in H_2O using 0.1% Formic acid as an additive. The fractions containing product were identified by LCMS and were combined, concentrated and freeze-dried to give **UOM-005447** (17.5 mg, 0.036 mmol, 20% yield) as a yellow powder. ^{1}H NMR (400 MHz, DMSO-d_6) δ ppm 1.51 (d, J=7.00 Hz, 3 H) 3.98 (s, 2 H) 4.11 (q, J=6.88 Hz, 1 H) 7.17 (d, J=8.00 Hz, 1 H) 7.22 (s, 1 H) 7.30 (dd, J=7.75, 4.75 Hz, 1 H) 7.36 (dd, J=8.57, 1.94 Hz, 1 H) 7.45 - 7.50 (m, 2 H) 7.57 (d, J=7.75 Hz, 1 H) 8.10 (d, J=8.13 Hz, 1 H) 8.16 (d, J=1.88 Hz, 1 H) 8.29 (d, J=1.88 Hz, 1 H) 8.41 (dd, J=4.69, 1.19 Hz, 1 H) 11.40 (s, 1 H) 12.45 (br. s., 1 H). LRMS (ESI) m/z $[M+H]^+$ calcd for $C_{24}H_{20}ClN_4OS_2$ 479.1, found 479.1.

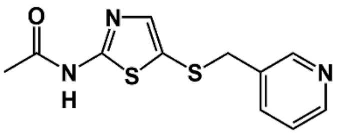

N-(5-((pyridin-3-ylmethyl)thio)thiazol-2-yl)acetamide(**UOM-005188**). Synthesized according to the procedure described for **II**. Purified 200 mg of crude to give **UOM-005188** (90.1 mg, 0.40 mmol, 45% yield) as a yellow powder. ^{1}H NMR (400 MHz, DMSO-d_6) δ ppm 2.12 (s, 3 H) 4.00 (s, 2 H) 7.24 (s, 1 H) 7.32 (dd, J=7.69, 4.82 Hz, 1 H) 7.59 (d, J=7.25 Hz, 1 H) 8.29 (s, 1 H) 8.42 (d, J=4.75 Hz, 1 H) 12.19 (br. s., 1 H). LRMS (ESI) m/z $[M+H]^+$ calcd for $C_{11}H_{12}N_3OS_2$ 266.0, found 266.0.

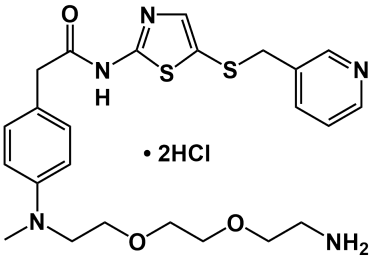

2-(4-((2-(2-(2-aminoethoxy)ethoxy)ethyl)(methyl)amino)phenyl)-N-(5-((pyridin-3-ylmethyl)thio)thiazol-2-yl)acetamide (**UOM-005790**). **Step 1:** This compound was synthesized using the general method for peptide coupling. **XVII** (59.2 mg, 0.325 mmol, 1.3 equiv.), 5-((pyridin-3-ylmethyl)thio)thiazol-2-amine (**I**) (55.8 mg, 0.25 mmol, 1.0 equiv.), DIPEA (200 µL, 1.12 mmol, 4.5 equiv.), and BTFFH (118.6 mg, 0.38 mmol, 1.5 equiv.). The reaction was dry loaded in celite and was purified in C18 EZ Prep using a gradient from 30% to 95% MeOH in H_2O using 0.1% Formic acid as an additive. The fractions containing product were identified by LCMS and were combined, concentrated and freeze-dried to give tert-butyl (2-(2-(2-(methyl(4-(2-oxo-2-((5-((pyridin-3-ylmethyl)thio)thiazol-2-yl)amino)ethyl)phenyl)amino)ethoxy)ethoxy)ethyl)carbamate (23.9 mg, 31% yield). ^{1}H NMR (400 MHz, $CDCl_3$) δ ppm 1.46 (s, 9 H) 3.01 (s, 3 H) 3.31 (q, J=4.79 Hz, 2 H) 3.51 - 3.63 (m, 8 H) 3.64 - 3.69 (m, 2 H) 3.71 (s, 2 H) 3.86 (s, 2 H) 4.99 (br. s., 1 H) 6.71 (d, J=8.63 Hz, 2 H) 7.08 (s, 1 H) 7.12 (d, J=8.63 Hz, 2 H) 7.22 (dd, J=7.82, 4.82 Hz, 1 H) 7.50 (dd, J=6.88, 2.50 Hz, 1 H) 8.32 (d, J=1.75 Hz, 1 H) 8.48 (d, J=3.63 Hz, 1 H) 8.90 (br. s., 1 H). **Step 2**: The previous intermediate, tert-butyl (2-(2-(2-(methyl(4-(2-oxo-2-((5-((pyridin-3-ylmethyl)thio)thiazol-2-yl)amino)ethyl)phenyl)amino)ethoxy)ethoxy)ethyl)carbamate (11.2 mg, 0.0186 mmol) was dissolved in DCM (1.00 mL) and added a 4 M solution of hydrochloric acid in dioxane (465 uL, 1.86 mmol, 100 equiv.). The reaction was stirred at r.t. for 30 min and upon completion of the reaction, the solvent was evaporated and the solid was suspended in heptane, sonicated, and filtered. The resulting solid was freeze dried to give **UOM-005790** (9.8 mg, 0.017 mmol, 92% yield) (29% yield over two steps) as a white solid. ^{1}H NMR (400 MHz, DMSO-d_6) δ ppm 2.90 - 3.00 (m, 4 H) 3.42 - 3.61 (m, 11 H) 3.63 - 3.74 (m, 5 H) 7.18 - 7.27 (m, 2 H) 7.28 (s, 1 H) 7.88 - 7.95 (m, 1 H) 7.98 (br. s., 3 H) 8.25 (s, 1 H) 8.65 (s, 1 H) 8.77 (d, J=5.38 Hz, 1 H) 12.53 (br. s., 1 H). LRMS (ESI) m/z $[M+H]^+$ calcd for $C_{24}H_{32}N_5O_3S_2$ 502.2, found 502.2.

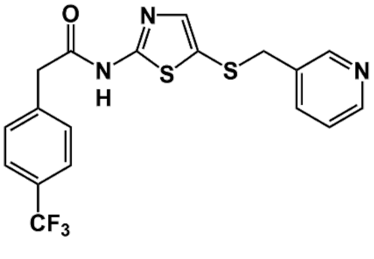

N-(5-((pyridin-3-ylmethyl)thio)thiazol-2-yl)-2-(4-(trifluoromethyl)phenyl)acetamide (**UOM-005203**). This compound was synthesized using the general method for peptide coupling. 4-(Trifluoromethyl)phenyl acetic acid (56.4 mg, 0.325 mmol, 1.3 equiv.), 5-((pyridin-3-ylmethyl)thio)thiazol-2-amine (**I**) (55.8 mg, 0.25 mmol, 1.0 equiv.), DIPEA (200 µL, 1.12 mmol, 4.5

equiv.), and BTFFH (118.6 mg, 0.38 mmol, 1.5 equiv.). The reaction was dry loaded in celite and was purified in C18 EZ Prep using a gradient from 30% to 95% MeOH in H_2O using 0.1% Formic acid as an additive. The fractions containing product were identified by LCMS and were combined, concentrated and freeze-dried to give **UOM-005203** (16.7 mg, 0.041 mmol, 16% yield). ^{1}H NMR (400 MHz, DMSO-d_6) δ ppm 3.89 (s, 2 H) 4.00 (s, 2 H) 7.27 (s, 1 H) 7.31 (dd, J=7.75, 4.75 Hz, 1 H) 7.53 (d, J=8.13 Hz, 2 H) 7.59 (d, J=7.88 Hz, 1 H) 7.70 (d, J=8.13 Hz, 2 H) 8.28 (br. s., 1 H) 8.42 (br. s., 1 H) 12.53 (s, 1 H). LRMS (ESI) m/z $[M+H]^+$ calcd for $C_{18}H_{15}F_3N_3OS_2$ 410.1, found 410.0.

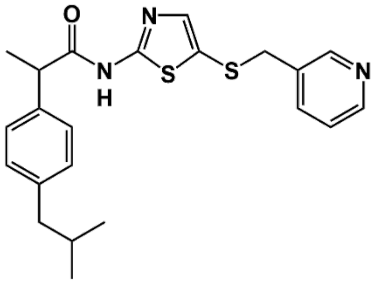

2-(4-isobutylphenyl)-N-(5-((pyridin-3-ylmethyl)thio)thiazol-2-yl)propenamide (**UOM-005430**). This compound was synthesized using the general method for peptide coupling. Ibuprofen free acid (48.0 mg, 0.233 mmol, 1.3 equiv.), 5-((pyridin-3-ylmethyl)thio)thiazol-2-amine (**I**) (40.0 mg, 0.179 mmol, 1.0 equiv.), DIPEA (156 µL, 0.896 mmol, 5.0 equiv.), and BTFFH (85.0 mg, 0.269 mmol, 1.5 equiv.). The reaction was dry loaded in celite and was purified in C18 EZ Prep using a gradient from 30% to 95% MeOH in H_2O using 0.1% Formic acid as an additive. The fractions containing product were identified by LCMS and were combined, concentrated and freeze-dried to give **UOM-005430** (33.6 mg, 0.082 mmol, 46% yield). ^{1}H NMR (400 MHz, DMSO-d_6) δ ppm 0.84 (d, J=6.63 Hz, 6 H) 1.40 (d, J=7.00 Hz, 3 H) 1.79 (dquin, J=13.47, 6.73, 6.73, 6.73, 6.73 Hz, 1 H) 2.40 (d, J=7.13 Hz, 2 H) 3.92 (q, J=7.00 Hz, 1 H) 3.99 (s, 2 H) 7.11 (d, J=8.13 Hz, 2 H) 7.22 (s, 1 H) 7.25 (d, J=8.00 Hz, 2 H) 7.31 (dd, J=7.82, 4.82 Hz, 1 H) 7.59 (dt, J=7.75, 1.88 Hz, 1 H) 8.29 (d, J=2.00 Hz, 1 H) 8.42 (dd, J=4.82, 1.56 Hz, 1 H) 12.39 (br. s., 1 H). LRMS (ESI) m/z $[M+H]^+$ calcd for $C_{22}H_{26}N_3OS_2$ 412.2, found 412.2.

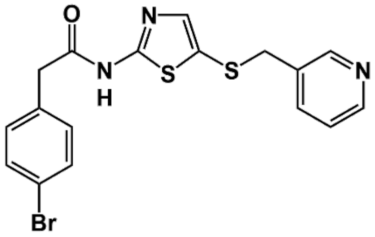

2-(4-bromophenyl)-N-(5-((pyridin-3-ylmethyl)thio)thiazol-2-yl)acetamide (**UOM-005199**). This compound was synthesized using the general method for peptide coupling. p-Bromophenylacetic acid (70.0 mg, 0.325 mmol, 1.3 equiv.), 5-((pyridin-3-ylmethyl)thio)thiazol-2-amine (**I**) (55.8 mg, 0.25 mmol, 1.0 equiv.), DIPEA (200 µL, 1.12 mmol, 4.5 equiv.), and BTFFH (118.6 mg, 0.38 mmol, 1.5 equiv.). The reaction was dry loaded in celite and was purified in C18 EZ Prep using a gradient from 30% to 95% MeOH in H_2O using 0.1% Formic acid as an additive. The fractions containing product were identified by LCMS and were combined, concentrated and freeze-dried to give **UOM-005199** (69.0 mg, 0.164 mmol, 66% yield) as a white powder. ^{1}H NMR (400 MHz, DMSO-d_6) δ ppm 3.75 (s, 2 H) 3.99 (s, 2 H) 7.24 - 7.28 (m, 3 H) 7.31 (dd, J=7.82, 4.82 Hz, 1 H) 7.52 (s, 2 H) 7.59 (dt, J=7.85, 1.89 Hz, 1 H) 8.28 (d, J=2.00 Hz, 1 H) 8.42 (dd, J=4.82, 1.56 Hz, 1 H) 12.47 (br. s., 1 H). LRMS (ESI) m/z $[M+H]^+$ calcd for $C_{17}H_{15}BrN_3OS_2$ 420.0, found 420.0.

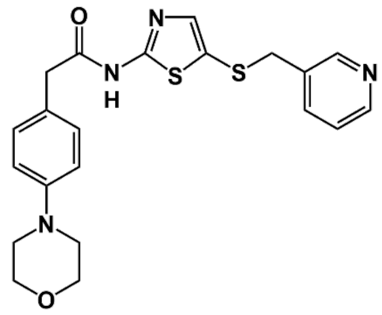

2-(4-morpholinophenyl)-N-(5-((pyridin-3-ylmethyl)thio)thiazol-2-yl)acetamide (**UOM-005606**). This compound was synthesized using the general method for peptide coupling. 2-(4-Morpholinophenyl)acetic acid(Lang *et al*, 2014) (27.3 mg, 0.124 mmol, 1.2 equiv.), 5-((pyridin-3-ylmethyl)thio)thiazol-2-amine (**I**) (23.0 mg, 0.103 mmol, 1.0 equiv.), DIPEA (81 µL, 0.463 mmol, 4.5 equiv.), and BTFFH (49.0 mg, 0.154 mmol, 1.5 equiv.). The reaction was concentrated and redissolved in DMSO and in prep-HPLC using a gradient from 40% to 95% MeOH in H_2O using 0.1% Formic acid as an additive. The fractions containing product were identified by LCMS and were combined, concentrated and freeze-dried to give **UOM-005606** (14.5 mg, 0.034 mmol, 33% yield). ^{1}H NMR (400 MHz, DMSO-d_6) δ ppm 2.97 - 3.15 (m, 4 H) 3.62 (s, 2 H) 3.67 - 3.77 (m, 4 H) 3.99 (s, 2 H) 6.89 (m, J=8.51 Hz, 2 H) 7.16 (m, J=8.50 Hz, 2 H) 7.21 - 7.39 (m, 2 H) 7.58 (d, J=7.88 Hz, 1 H) 8.28 (s, 1 H) 8.42 (d, J=4.38 Hz, 1 H) 12.39 (br. s., 1 H). LRMS (ESI) m/z $[M+H]^+$ calcd for $C_{21}H_{23}N_4O_2S_2$ 427.1, found 427.1.

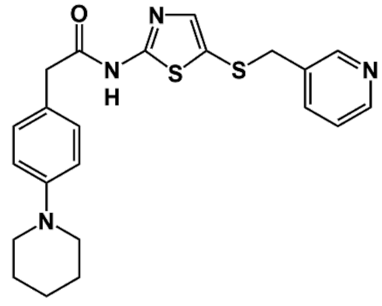

2-(4-(piperidin-1-yl)phenyl)-N-(5-((pyridin-3-ylmethyl)thio)thiazol-2-yl)acetamide (**UOM-005607**). This compound was synthesized using the general method for peptide coupling. 2-(4-(Piperidin-1-yl)phenyl)acetic acid(Hicks *et al*, 1979) (20.0 mg, 0.091 mmol, 1.2 equiv.), 5-((pyridin-3-ylmethyl)thio)thiazol-2-amine (**I**) (17.0 mg, 0.076 mmol, 1.0 equiv.), DIPEA (60 µL, 0.343 mmol, 4.5 equiv.), and BTFFH (36.1 mg, 0.114 mmol, 1.5 equiv.). The reaction was concentrated and redissolved in DMSO and in prep-HPLC using a gradient from 40% to 95% MeOH in H_2O using 0.1% Formic acid as an additive. The fractions containing product were identified by LCMS and were combined, concentrated and freeze-dried to give **UOM-005607** (5.5 mg, 0.013 mmol, 17% yield). ^{1}H NMR (400 MHz, DMSO-d_6) δ ppm 1.59 (d, J=4.63 Hz, 6 H) 3.01 - 3.20 (m, 4 H) 3.60 (s, 2 H) 3.99 (s, 2 H) 6.87 (m, J=8.38 Hz, 2 H) 7.12 (m, J=8.38 Hz, 2 H) 7.25 (s, 2 H) 8.29 (s, 2 H) 12.38 (s, 1 H). LRMS (ESI) m/z $[M+H]^+$ calcd for $C_{22}H_{25}N_4OS_2$ 425.1, found 425.1.

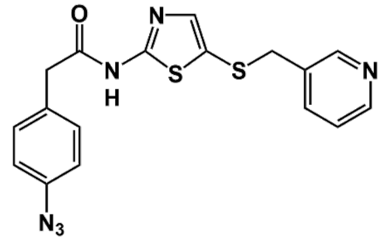

2-(4-azidophenyl)-N-(5-((pyridin-3-ylmethyl)thio)thiazol-2-yl)acetamide (**UOM-005062**). In a microwave vial, dissolve 2-(4-azidophenyl)acetic acid (50 mg, 0.282 mmol, 1.0 equiv.) in DCM (1.5 mL) and place in an ice bath. Separately dissolve oxalyl chloride (39.4 mg, 0.310 mmol, 1.1 equiv.) in DCM (1.5 mL) and add this solution to the carboxylic acid one. Stir the mixture at 0°C for 1h and then concentrate the mixture in the rotavap and place it under vacuum for 10 min. Then redissolve the mixture in DCM (1.5 mL) and add pyridine (70.0 mg, 0.847 mmol, 3.0 equiv.) and finally add 5-((pyridin-3-ylmethyl)thio)thiazol-2-amine (**I**) (69.3 mg, 0.310 mmol, 1.1 equiv.). Stir the reaction at r.t. under a nitrogen atmosphere overnight. Dry load the crude on celite and purified in C18 EZ Prep using a gradient from 30% to 95% MeOH in H_2O using 0.1% Formic acid as an additive. The fractions containing product were identified by LCMS and were combined, concentrated and freeze-dried to give **UOM-005062** (14.0 mg, 0.037

mmol, 13% yield). ^{1}H NMR (600 MHz, DMSO-d_6) δ ppm 3.75 (s, 2 H) 3.99 (s, 2 H) 7.07 - 7.11 (m, 2 H) 7.26 (s, 1 H) 7.31 (dd, J=7.89, 4.97 Hz, 1 H) 7.33 - 7.36 (m, 2 H) 7.59 (dt, J=7.75, 1.97 Hz, 1 H) 8.28 (d, J=1.75 Hz, 1 H) 8.42 (dd, J=4.68, 1.75 Hz, 1 H) 12.46 (br. s., 1 H). LRMS (ESI) m/z $[M+H]^+$ calcd for $C_{17}H_{15}N_6OS_2$ 383.1, found 383.1.

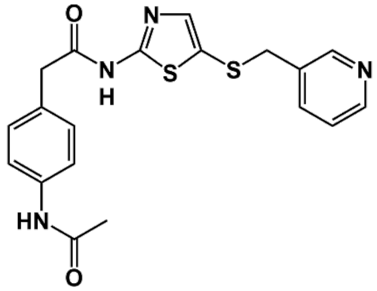

2-(4-acetamidophenyl)-N-(5-((pyridin-3-ylmethyl)thio)thiazol-2-yl)acetamide (**UOM-005473**). Dissolve **XIV** (30.0 mg, 0.0842 mmol, 1.0 equiv.) in a mixture of DCM (1.00 mL) and pyridine (200 µL). Add acetic anhydride (23.9 µL, 0.252 mmol) and stir the reaction overnight at r.t. The reaction was concentrated and redissolved in DMSO and purified in prep-HPLC using a solvent system from 35% to 95% MeOH in H_2O. The fractions containing product were combined and concentrated, then lyophilized to give **UOM-005473** (26.2 mg, 0.066 mmol, 78 %) as a white solid. ^{1}H NMR (400 MHz, DMSO-d_6) δ ppm 2.02 (s, 3 H) 3.68 (s, 2 H) 3.99 (s, 2 H) 7.21 (d, J=8.38 Hz, 2 H) 7.25 (s, 1 H) 7.31 (dd, J=7.75, 4.75 Hz, 1 H) 7.51 (d, J=8.38 Hz, 2 H) 7.58 (d, J=7.75 Hz, 1 H) 8.28 (s, 1 H) 8.42 (d, J=4.63 Hz, 1 H) 9.91 (s, 1 H) 12.42 (br. s., 1 H). LRMS (ESI) m/z $[M+H]^+$ calcd for $C_{19}H_{19}N_4O_2S_2$ 399.1, found 399.1.

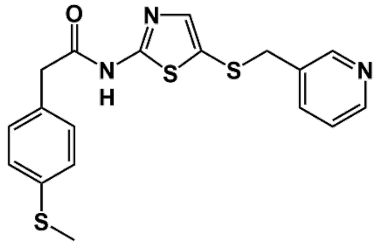

2-(4-(methylthio)phenyl)-N-(5-((pyridin-3-ylmethyl)thio)thiazol-2-yl)acetamide (**UOM-005206**). This compound was synthesized using the general method for peptide coupling. 4-(Methylthio)phenylacetic acid (59.2 mg, 0.325 mmol, 1.3 equiv.), 5-((pyridin-3-ylmethyl)thio)thiazol-2-amine (**I**) (55.8 mg, 0.25 mmol, 1.0 equiv.), DIPEA (200 µL, 1.12 mmol, 4.5 equiv.), and BTFFH (118.6 mg, 0.38 mmol, 1.5 equiv.). The reaction was dry loaded in celite and was purified in C18 EZ Prep using a gradient from 30% to 95% MeOH in H_2O using 0.1% Formic acid as an additive. The fractions containing product were identified by LCMS and were combined, concentrated and freeze-dried to give **UOM-005206** (80.8 mg, 0.208 mmol, 83.4% yield). ^{1}H NMR (400 MHz, DMSO-d_6) δ ppm 2.45 (s, 3 H) 3.71 (s, 2 H) 3.99 (s, 2 H) 7.23 (d, J=3.50 Hz, 3 H) 7.25 - 7.28 (m, 2 H) 7.31 (m, J=7.75 Hz, 1 H) 7.57 - 7.61 (m, 1 H) 8.28 (d, J=1.50 Hz, 1 H) 8.42 (m, J=3.25 Hz, 1 H) 12.46 (br. s., 1 H). LRMS (ESI) m/z $[M+H]^+$ calcd for $C_{18}H_{18}N_3OS_3$ 388.1, found 388.1.

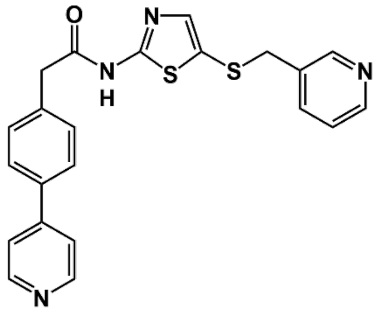

N-(5-((pyridin-3-ylmethyl)thio)thiazol-2-yl)-2-(4-(pyridin-4-yl)phenyl)acetamide (**UOM-005550**). **UOM-005200** (45.0 mg, 0.097 mmol, 1.0 equiv.) and Bis(triphenylphosphine)palladium(II) chloride (0.05 eq, 3.4 mg, 0.0048 mmol, 0.05 equiv.) were dissolved in dioxane (3 mL). Stirred at r.t for 5 min and then added pyridine-4-boronic acid (23.7 mg, 0.193 mmol, 2.0 equiv.) was added, followed by an aqueous solution of Na_2CO_3 (0.192 mL, 0.385 mmol, 4.0 equiv.). The mixture was sonicated for 2 min and then stirred overnight at 100°C for. Cooled the reaction to r.t., added water (0.5ml) and EtOAc (5ml), sonicated a bit, and filtered off the insoluble material over celite. Concentrated to nearly dryness, added DMSO (1.4ml) and 0.75 µL of formic acid. Purified

by Prep-HPLC and the fractions containing product were identified by LCMS and were combined, concentrated and freeze-dried to give **UOM-005550** (5.2 mg, 0.012 mmol, 13% yield) as an off-white powder. ^{1}H NMR (400 MHz, DMSO-d_6) δ ppm 3.83 (s, 2 H) 4.00 (s, 2 H) 7.17 - 7.39 (m, 2 H) 7.46 (m, J=8.25 Hz, 2 H) 7.59 (d, J=7.88 Hz, 1 H) 7.70 (d, J=6.00 Hz, 2 H) 7.77 (m, J=8.25 Hz, 2 H) 8.28 (s, 1 H) 8.41 (br. s., 1 H) 8.52 - 8.77 (m, 2 H). LRMS (ESI) m/z $[M+H]^+$ calcd for $C_{22}H_{19}N_4OS_2$ 419.1, found 419.1.

References

Currie KS, Kropf JE, Lee T, Blomgren P, Xu J, Zhao Z, Gallion S, Whitney JA, Maclin D, Lansdon EB, *et al* (2014) Discovery of GS-9973, a selective and orally efficacious inhibitor of spleen tyrosine kinase. *J Med Chem* 57: 3856–3873

Due-Hansen ME, Pandey SK, Christiansen E, Andersen R, Hansen SVF & Ulven T (2016) A protocol for amide bond formation with electron deficient amines and sterically hindered substrates. *Org Biomol Chem* 14: 430–433

Hicks TA, Smith CE, Williamson WR & Day EH (1979) Potential antiinflammatory compounds. 1. Antiinflammatory phenylpiperidine derivatives. *J Med Chem* 22: 1460–1464

Kim KS, Kimball SD, Misra RN, Rawlins DB, Hunt JT, Xiao H-Y, Lu S, Qian L, Han W-C, Shan W, *et al* (2002) Discovery of aminothiazole inhibitors of cyclin-dependent kinase 2: synthesis, X-ray crystallographic analysis, and biological activities. *J Med Chem* 45: 3905–3927

Lang SB, O’Nele KM & Tunge JA (2014) Decarboxylative allylation of amino alkanoic acids and esters via dual catalysis. *J Am Chem Soc* 136: 13606–13609
