# Supplementary figures and images for: DDB1 engagement defines the selectivity of S656 analogs for cyclin K degradation over CDK inhibition
Source: EMBO Rep. 2025 Apr 28;26(11):2836–54. doi: 10.1038/s44319-025-00448-y (PMC12152147; doi:10.1038/s44319-025-00448-y)

## Slide 1
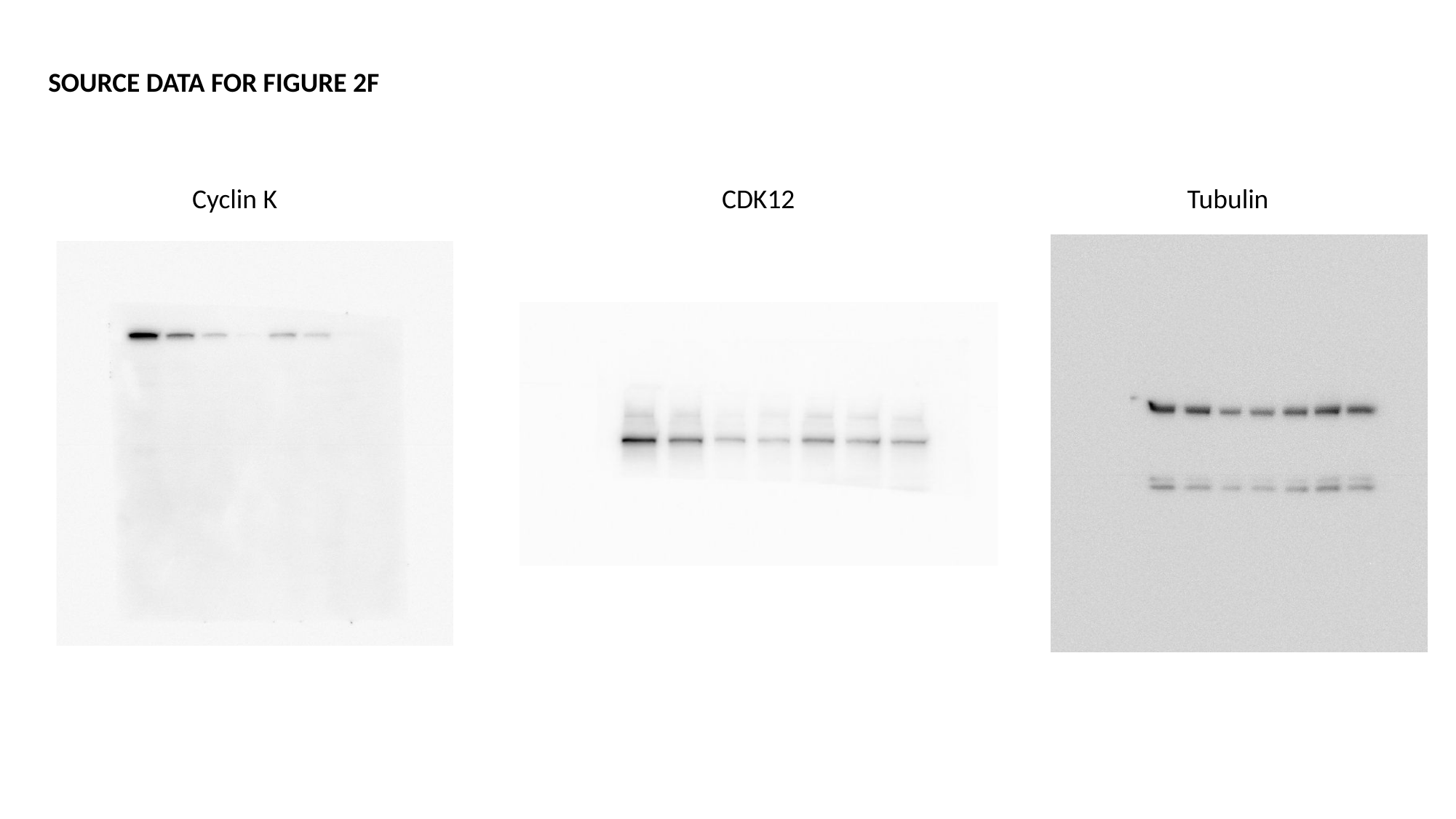

SOURCE DATA FOR FIGURE 2F
Cyclin K
CDK12
Tubulin

Supplement: Supplementary file 10 — Source data Fig. 2 [file 44319_2025_448_MOESM10_ESM.zip › Figure 2/2F/Figure 2F.pptx]

## Slide 1
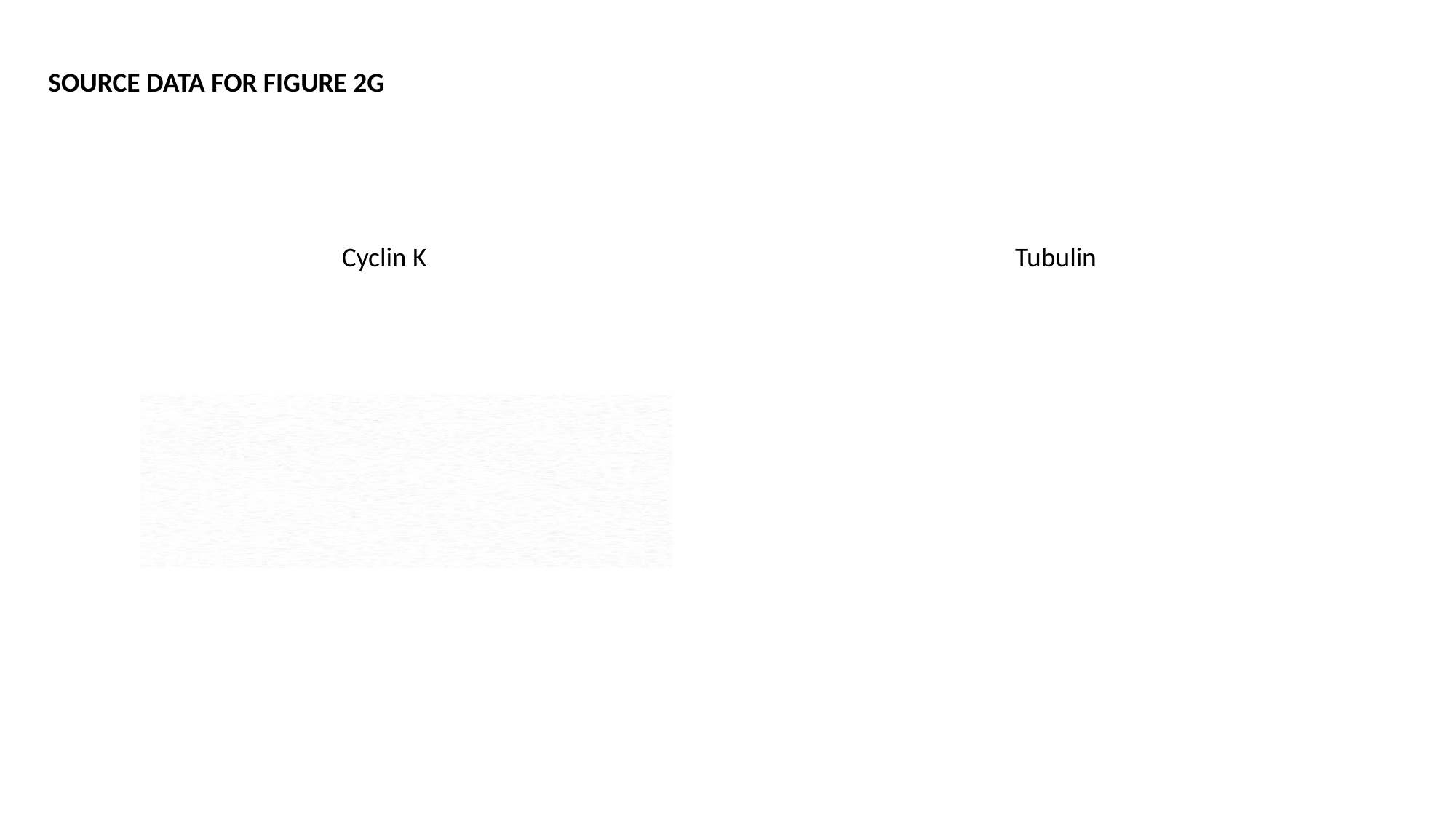

SOURCE DATA FOR FIGURE 2G
Cyclin K
Tubulin

Supplement: Supplementary file 10 — Source data Fig. 2 [file 44319_2025_448_MOESM10_ESM.zip › Figure 2/2G/Figure 2G.pptx]

## Slide 1
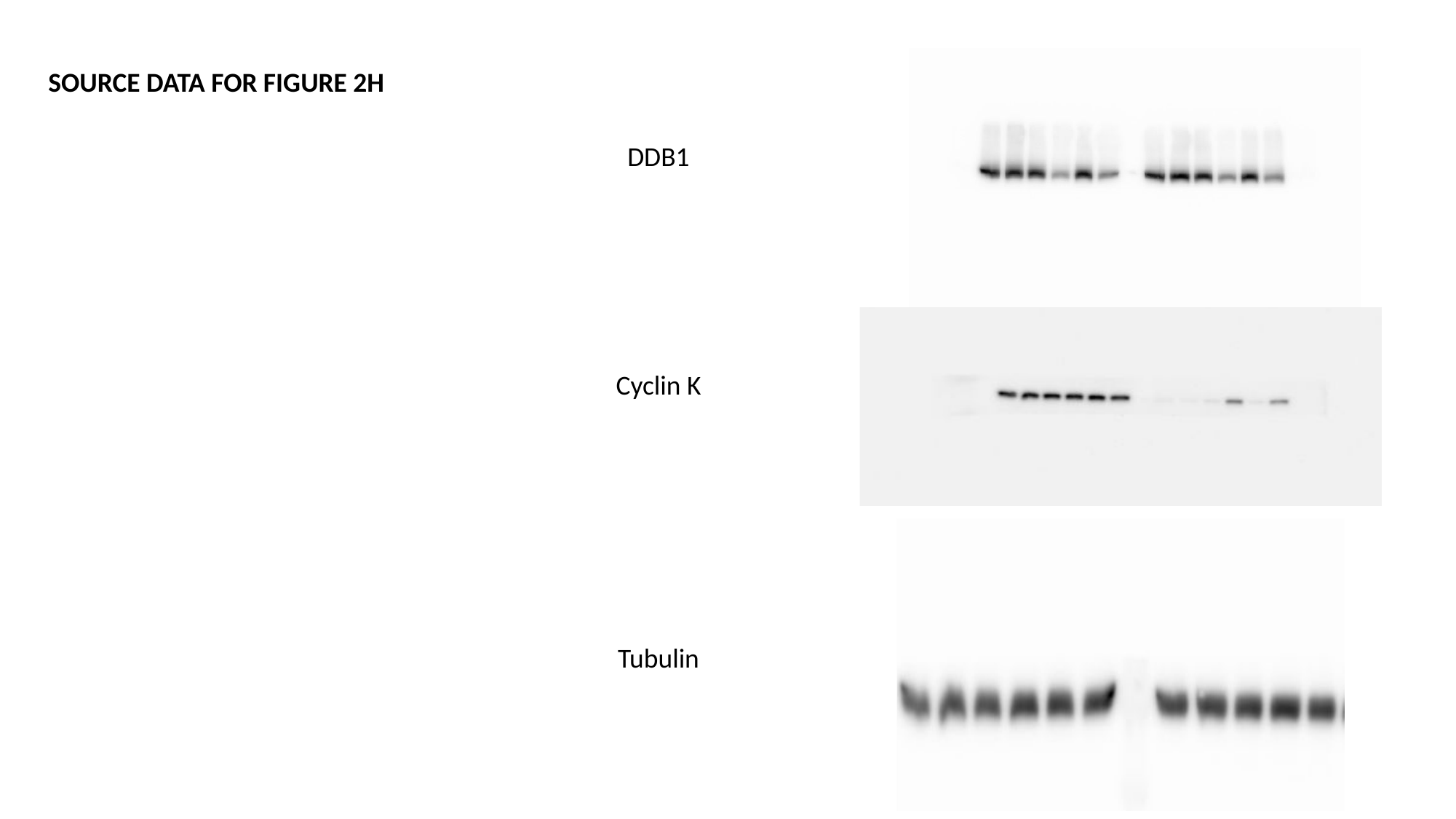

SOURCE DATA FOR FIGURE 2H
DDB1
Cyclin K
Tubulin

Supplement: Supplementary file 10 — Source data Fig. 2 [file 44319_2025_448_MOESM10_ESM.zip › Figure 2/2H/Figure 2H.pptx]

## Slide 1
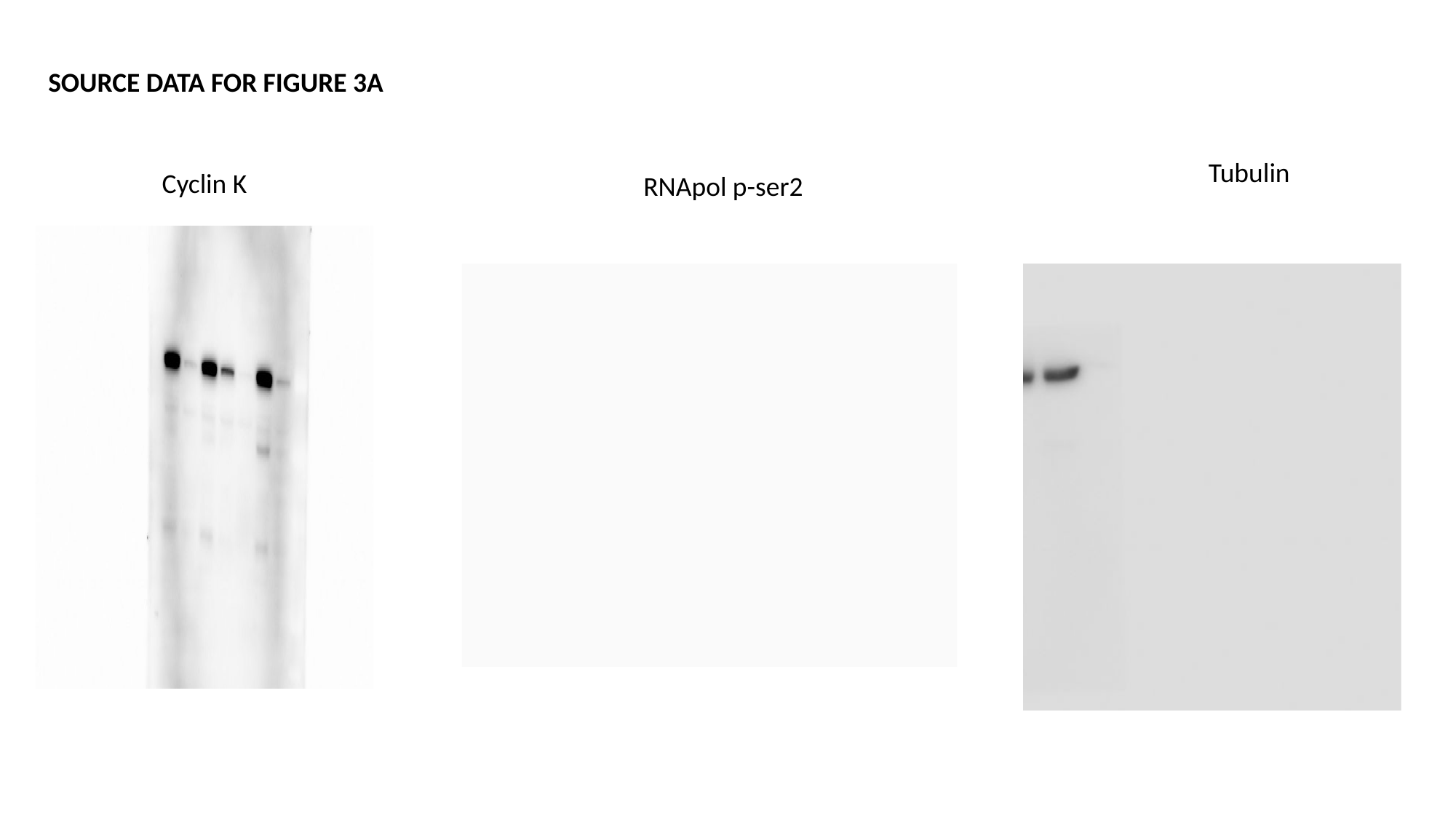

SOURCE DATA FOR FIGURE 3A
Tubulin
Cyclin K
RNApol p-ser2

Supplement: Supplementary file 11 — Source data Fig. 3 [file 44319_2025_448_MOESM11_ESM.zip › Figure 3/3A/Figure 3A.pptx]

## Slide 1
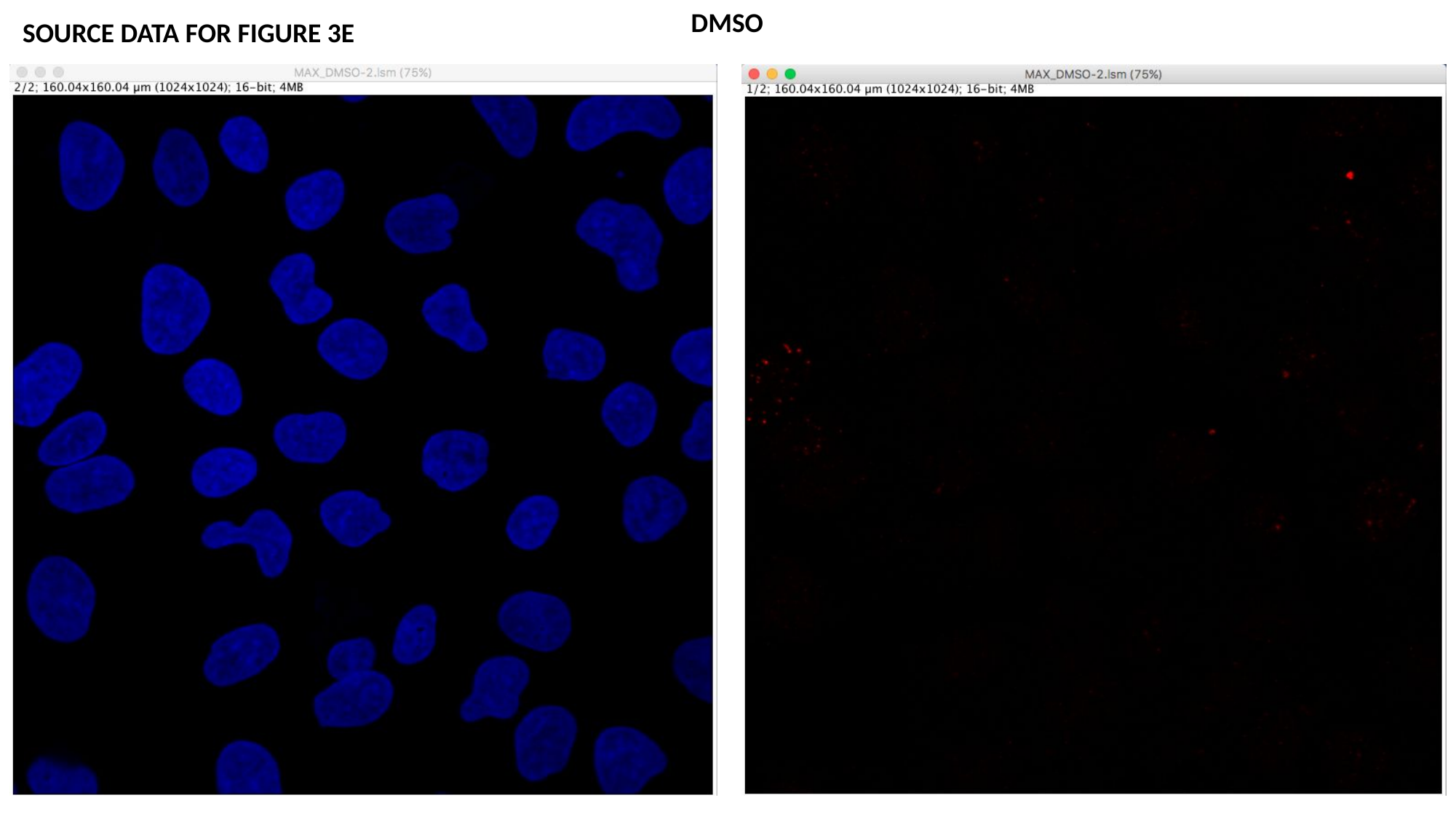

DMSO
SOURCE DATA FOR FIGURE 3E

## Slide 2
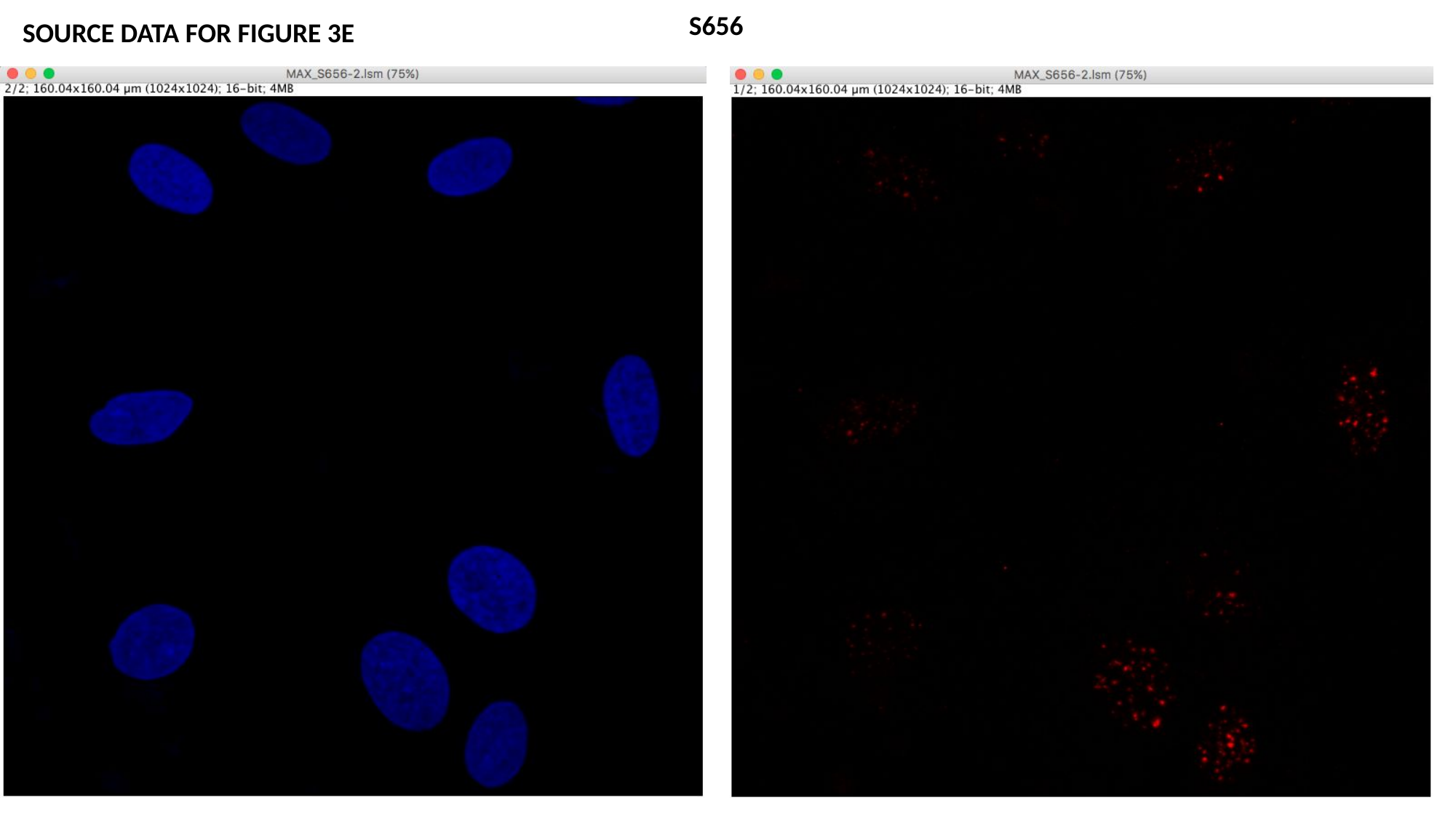

S656
SOURCE DATA FOR FIGURE 3E

Supplement: Supplementary file 11 — Source data Fig. 3 [file 44319_2025_448_MOESM11_ESM.zip › Figure 3/3E/Figure 3E.pptx]

## Slide 1
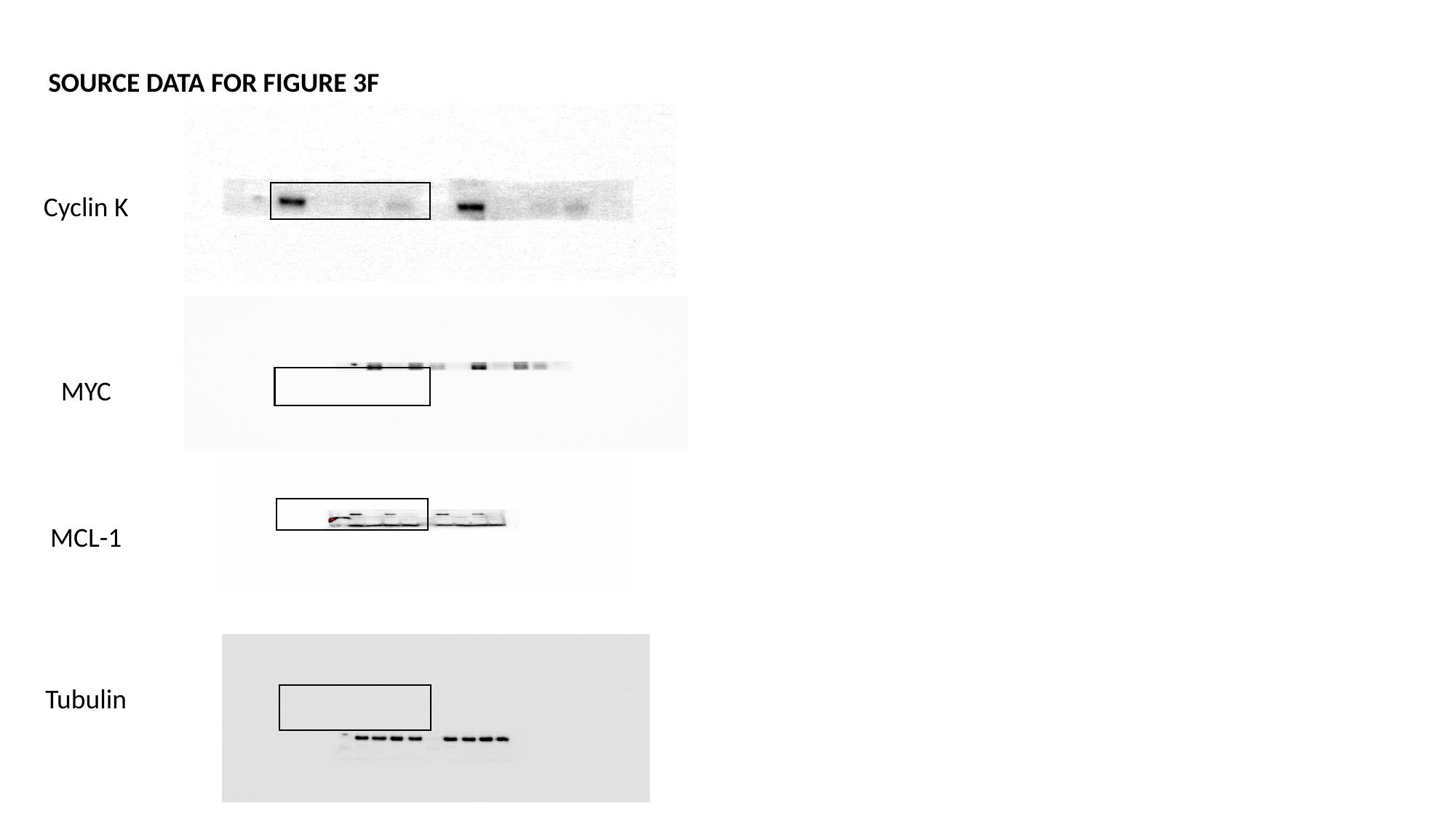

SOURCE DATA FOR FIGURE 3F
Cyclin K
MYC
MCL-1
Tubulin

Supplement: Supplementary file 11 — Source data Fig. 3 [file 44319_2025_448_MOESM11_ESM.zip › Figure 3/3F/Figure 3F.pptx]

## Slide 1
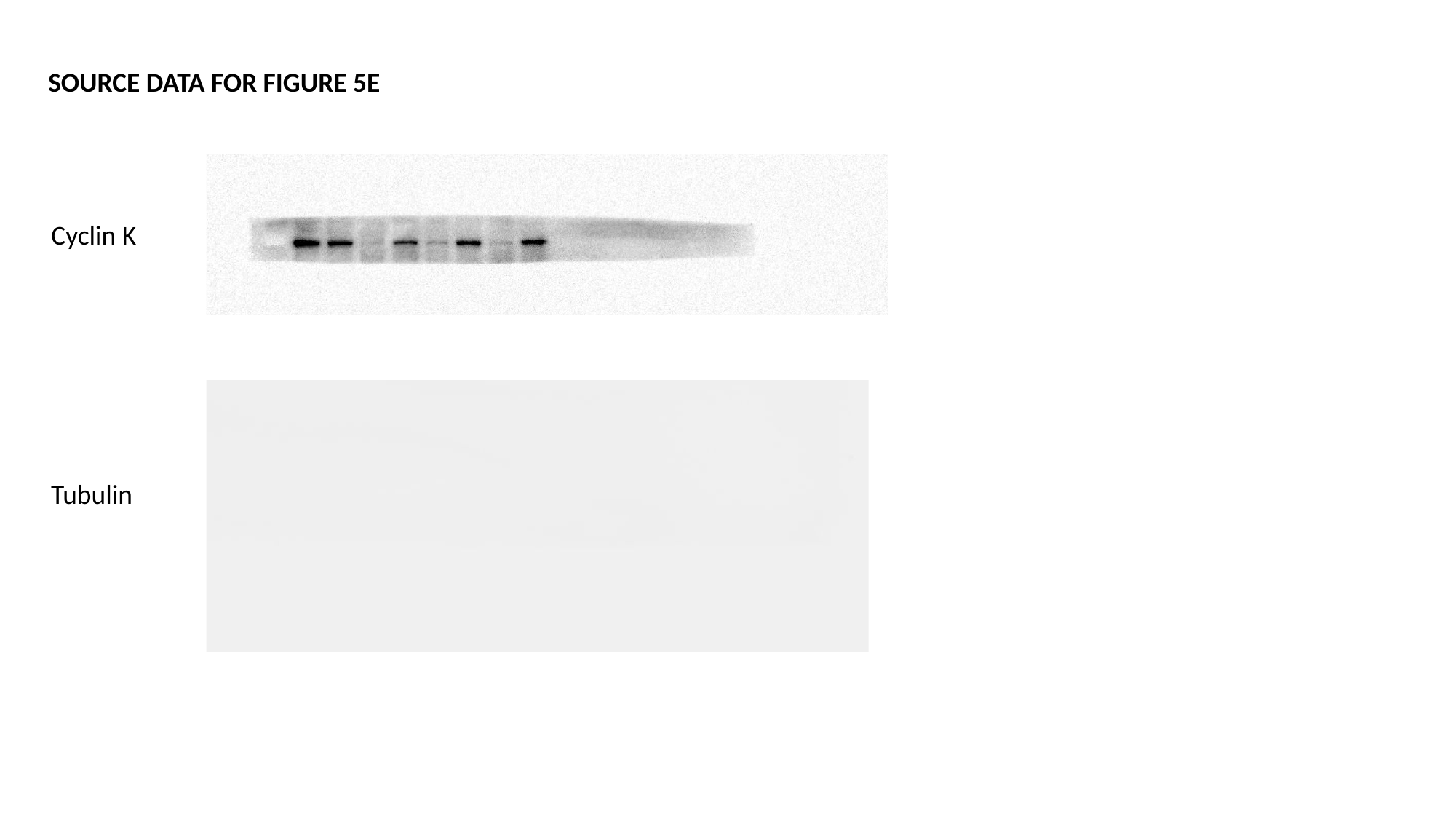

SOURCE DATA FOR FIGURE 5E
Cyclin K
Tubulin

Supplement: Supplementary file 13 — Source data Fig. 5 [file 44319_2025_448_MOESM13_ESM.zip › Figure 5/5E/Figure 5E.pptx]
